# Wideband mmWave antenna with integrated 1 × 8 feed-array configuration for high-gain 5G wireless applications using characteristic mode theory

**DOI:** 10.1038/s41598-026-68057-w

**Published:** 2026-08-29

**Authors:** Parveez Shariff B. G., Hüseyin Şerif Savci, Hela Elmannai, Aishah Alsehaim, Altaf Ahmed Mugheri, Saad Hassan Kiani, Mohd Imran Ibrahim, Mariana Dalarsson

**Affiliations:** 1 Department of Electronics and Communication Engineering, Dayananda Sagar University, Bengaluru, 562112 India; 2 Electrical and Electronics Engineering Department, RF Microsense Research Group, Istanbul Medipol University, 34810 Istanbul, Turkey; 3 Department of Information Technology, College of Computer and Information Sciences, Princess Nourah Bint Abdulrahman University, P.O. Box 84428, 11671 Riyadh, Saudi Arabia; 4 Faculty of Electronics and Computer Engineering, Universiti Teknikal Melaka Malaysia, 7600 Melaka, Malaysia; 5 School of Electrical Engineering and Computer Science, KTH Royal Institute of Technology, 100 − 44 Stockholm, Sweden

**Keywords:** 28 GHz, 38 GHz, CMT, High gain, Integrated feed network array, Linear array antennas, Engineering, Physics

## Abstract

This work presents a wideband millimeter-wave (mmWave) antenna designed and experimentally validated using the Characteristic Mode Theory (CMT). The proposed single-element is implemented on an ultra-thin 0.254 mm substrate and occupies a compact footprint of 8 × 6 mm^2^. The antenna achieves a wide impedance bandwidth from 22.5 to 39 GHz, corresponding to a fractional bandwidth of 53.66%, covering the major 28 GHz and 38 GHz 5G mmWave bands. CMT analysis is employed to investigate the dominant electric-dipole modes across various frequency bands. Further, by keeping the ground length constant at $0.3{\lambda}_{0}$, the monople structure is varied to suppress the magnetic-dipole modes, thereby improving the linear polarization behavior with electric-dipole modes. The CMT for the proposed single-element structure illustrates that modes ${J}_{3}+{J}_{1}+{J}_{4}$, ${J}_{3}+{J}_{4}+{J}_{5}$, ${J}_{3}+{J}_{2}+{J}_{4}$, and ${J}_{2}+{J}_{3}+{J}_{5}$ are contributing to resonance at 24, 28, 32, and 36 GHz, where the first modes in each band indicate the dominant mode with relatively higher magnitude. The summation of these multiple excited modes across multiple bands has led to a wideband resonance with a stable radiation pattern. To improve the radiation performance for high-gain mmWave applications, the optimized single element is extended into a 1 × 8 linear array configuration. The proposed array achieves a gain variation from 11 to 15.8 dBi across the operating band, with peak gains of 12.8 dBi at 28 GHz and 15.5 dBi at 38 GHz. The measured results obtained from the developed prototype show good agreement with the simulated responses, validating the proposed CMT-guided design approach. Due to its compact size, ultra-thin profile, wide bandwidth, high-gain array performance, and clear modal interpretation, the proposed antenna is a promising candidate for future 5G mmWave wireless communication systems.

## Introduction

Fifth-generation (5G) wireless communication has been developed to support the rapidly increasing demand for high data rates, low latency, massive connectivity, and reliable wireless services^1,2^. Modern applications such as high-definition video streaming, cloud computing, Internet of Things (IoT), smart transportation, industrial automation, and real-time communication require larger bandwidth and improved network capacity^3^. In early 5G deployment, sub-6 GHz frequency bands have been widely adopted because they provide reliable coverage, good penetration through buildings, and stable connectivity over longer distances^4^. These bands are suitable for wide-area mobile communication due to their lower propagation loss and better non-line-of-sight performance^5–7^. In addition, sub-6 GHz systems benefit from mature infrastructure, cost-effective deployment, and good compatibility with existing wireless devices. However, the limited available bandwidth in the sub-6 GHz spectrum restricts the achievable data rate and capacity, especially in dense urban networks with rapidly increasing user traffic^8,9^.

To overcome these limitations, millimeter-wave (mmWave) communication has become an important enabling technology for 5G and future wireless systems^10,11^. The mmWave spectrum offers abundant bandwidth, enabling very high data rates, low latency, and high-capacity wireless links. In practical 5G systems, frequency bands around 24, 26, 28, 37, 38, and 39 GHz have received significant attention for enhanced mobile broadband, fixed wireless access, high-capacity backhaul, and short-range high-speed communication^12,13^. However, mmWave signals suffer from higher free-space path loss, atmospheric attenuation, penetration loss, and greater sensitivity to blockage than lower-frequency signals. These limitations reduce the communication range and make the wireless link more sensitive to obstacles such as buildings, walls, human bodies, and foliage^14^. Therefore, antenna design plays a critical role in mmWave systems by providing wide bandwidth, high gain, stable radiation patterns, compact size, and efficient radiation performance. Since the wavelength at mmWave frequencies is very small, compact antenna elements and array configurations can be realized within a limited area. Wideband and high-gain antennas are especially important because they can support multiple mmWave bands while improving link reliability through enhanced directivity^15,16^. In this context, physically explainable design methods, such as characteristic mode analysis, are useful because they provide deeper insight into modal behavior and radiation mechanisms, rather than relying solely on conventional full-wave optimization^17^.

In the literature, several wideband and feed-network arrays have been proposed^18–26^. A broadband wire-hexagon antenna array at 28 GHz was presented in^18^. The single radiating element consisted of two concentric hexagonal wire loops printed on the front side, while the backside included a truncated ground plane extended into a larger hexagonal ground loop to improve broadside radiation. An inverted U-shaped via wall was also introduced around the monopole to stabilize the inter-element coupling and enhance the array performance. The array occupied a compact footprint of 45 × 20 mm^2^ and achieved a wide operating band from 25.052 to 34.923 GHz, corresponding to a bandwidth of 9.871 GHz. The antenna array provided a peak gain of 12.15 dBi, radiation efficiency above 85%, and narrow beamwidths of 44.3° and 12.5° in the xz- and yz-planes, respectively. In^19^, a bowtie structure with a four-element feed network array using a corporate microstrip feed network was presented. The array occupied a compact size of 37.6 × 14.3 mm^2^ and achieved a wide operating bandwidth from 23.41 to 33.92 GHz, corresponding to 35.53% fractional bandwidth at 28 GHz. A peak gain of 10.7 dBi and radiation efficiency higher than 90% across the operating band. A narrow beamwidth of 14.6° was obtained in the H-plane, making the design suitable for directional mmWave links. Similarly, a snowflake antenna array with dual-beam characteristics was proposed in^20^ at 28 GHz. The symmetric snowflake configuration enabled stable dual-beam radiation over the operating bandwidth, with two beams located around ± 50° in the xy-plane at 28 GHz. To enhance gain, the antenna was extended into a 4-element array with an overall size of 32 × 12 mm^2^. The array achieved a gain of about 10.15 dBi at 28 GHz, with efficiency above 80%. However, the array bandwidth reduced significantly to 27.45–28.86 GHz, which is much narrower than the single-element response. Moreover, the array beam behavior changed from the original ± 50° dual-beam response to two opposite beams at 0° and 180°, limiting beam-direction flexibility. A planar, wideband, circularly polarized, cavity-backed 4 × 4 stacked patch array was reported in^21^ for millimeter-wave applications. The antenna was implemented with two substrate layers: a driven patch layer and a parasitic patch layer. To achieve wideband circular polarization, the design integrated an SIW cavity-backed structure, a CPW feeding network, and a sequential-rotation feeding network. The array achieved a wide impedance bandwidth of 25.06–33.76 GHz and a peak gain of 20.32 dBic at 30.5 GHz. Although the antenna provides high-gain, wideband, circularly polarized performance, its stacked multilayer configuration, SIW cavity, and combined SIW–CPW feeding arrangement increase overall design complexity and fabrication sensitivity. Moreover, grating-lobe behavior was observed in the radiation patterns due to the relatively large spacing between the driven patches. In^22^, a compact asymmetric dipole array was presented for broadband mmWave portable applications. The design consists of eight asymmetric dipole elements arranged in a 1-D linear array and implemented on a single-layer FR-4 substrate. Unlike conventional tightly coupled dipole arrays that often require baluns, impedance transformers, or decoupling circuits, this structure achieves wideband operation without additional auxiliary feeding or isolation networks. The antenna uses strong inter-element coupling and leaky-wave radiation behavior to obtain wide-angle beam coverage over the 25–35 GHz band. The compact array provides more than ± 70° beam scanning from 25 to 29 GHz and more than ± 55° beam scanning from 31 to 35 GHz. Although the design offers wide scanning coverage and low-cost fabrication, using FR-4 at mmWave frequencies may increase dielectric loss, and the work primarily focuses on beam coverage rather than high-gain array enhancement. In^23^, a compact metasurface-based 1 × 2 antenna array was presented, in which rectangular slots were introduced into the radiating patches to improve impedance matching and bandwidth. To further enhance the radiation performance, a single-layer reflecting metasurface consisting of a 2 × 4 arrangement of diamond-shaped split-ring unit cells was positioned approximately at half operating wavelength above the antenna array, forming a Fabry–Perot cavity. The proposed structure achieved an operating bandwidth of 27.38–33.34 GHz, corresponding to 5.96 GHz bandwidth, with a maximum gain of about 12.7 dBi. The overall physical size of the antenna was 20.5 × 20.6 × 0.508 mm^3^. Although the design provides high gain, wide bandwidth, and good efficiency, the use of a suspended metasurface layer increases the overall antenna profile and adds alignment sensitivity between the metasurface and the driven array.

A 4 × 4 planar array of X-shaped slot antennas for 28 GHz is presented in^24^. The X-shaped slot element was developed from a leaf-shaped bowtie slot antenna and used as the radiating element of the planar array. The antenna employed microstrip and slot-line feeding, while a flat reflector was placed below the array to obtain unidirectional broadside radiation. To further enhance gain and bandwidth, two dielectric superstrate layers were introduced above the 4 × 4 array, creating resonance cavities between the ground plane and the superstrate layers. The optimized antenna achieved impedance bandwidths of 25.35–25.95 GHz and 26.8–30.2 GHz, with realized gain ranging from 17.5 to 20 dBi across the operating bands. The design offers high gain and good bandwidth performance around the 28 GHz 5G band. However, the use of a reflector and two superstrate layers increases the overall antenna profile and structural complexity. In^25^, a millimeter-wave beam-scanning antenna array was presented using collocated multiple-radiating-mode microstrip patch elements. The design consists of a four-element linear array operating around 24.3 GHz with an overall size of 61.2 × 28.2 mm^2^. Each element uses a dual-mode circular patch configuration, where the inner circular patch excites the broadside TM11 mode,s and the outer shorted circular patch excites the conical TM21 mode. The combination of these two modes provides additional design freedom for reducing grating lobes and sidelobe levels, even with a large inter-element spacing of approximately one wavelength. In^26^, a high-gain endfire 1 × 8 linear array was reported using a stripline-fed radiating-via monopole structure. In this design, each element uses a vertically oriented radiating via directly excited through a stripline feed, while surrounding ground vias and metallic plates are used to confine the electromagnetic fields, suppress surface-wave leakage, and support stable endfire radiation. The array occupies a compact size of 34.1 × 47.6 mm^2^ and operates from 24 to 30.12 GHz, corresponding to a fractional bandwidth of 22.62%.

Unlike reported mmWave arrays that primarily rely on conventional array optimization, metasurface loading, stacked substrates, superstrates, cavity-backed structures, or complex feeding arrangements, this work introduces a CMT-guided wideband mmWave antenna with an integrated 1 × 8 feed array. Recent literature suggests that the CMT is used to generate orthogonal modes for dual-band operation^27^, decoupling, and pattern correction^28^, quad-modes for circular polarization^29^, and many more. However, in this article, the CMT is used to excite multiple electric dipole modes across multiple frequency bands, thereby suppressing the magnetic dipole and achieving wideband characteristics with linear polarization. The main strength of this work is that the antenna is not only optimized for wideband operation, but its radiation behavior is physically explained through characteristic mode analysis. The proposed single-element antenna occupies only 8 × 6 mm^2^ on an ultra-thin 0.254 mm substrate and achieves a wide bandwidth of 22.5–39 GHz, covering both the 28 GHz and 38 GHz 5G mmWave bands. The CMT results show that Modes ${J}_{3}+{J}_{1}+{J}_{4}$ dominates at 24 GHz, ${J}_{3}+{J}_{4}+{J}_{5}$ dominates at 28 GHz, ${J}_{3}+{J}_{2}+{J}_{4}$ dominates 34 GHz, and ${J}_{2}+{J}_{3}+{J}_{5}$ dominates 38 GHz, which explains the wideband resonance mechanism. Furthermore, the transformation of the single element into a 1 × 8 feed-array improves the gain to 11–15.8 dBi across the operating band. Hence, the proposed antenna combines compact size, ultra-thin profile, wide dual-band mmWave coverage, high-gain array performance, and modal-based design interpretation, making it a technically strong candidate for future high-gain 5G mmWave wireless systems.

## Design methodology

### Single-element antenna

**Fig. 1:**
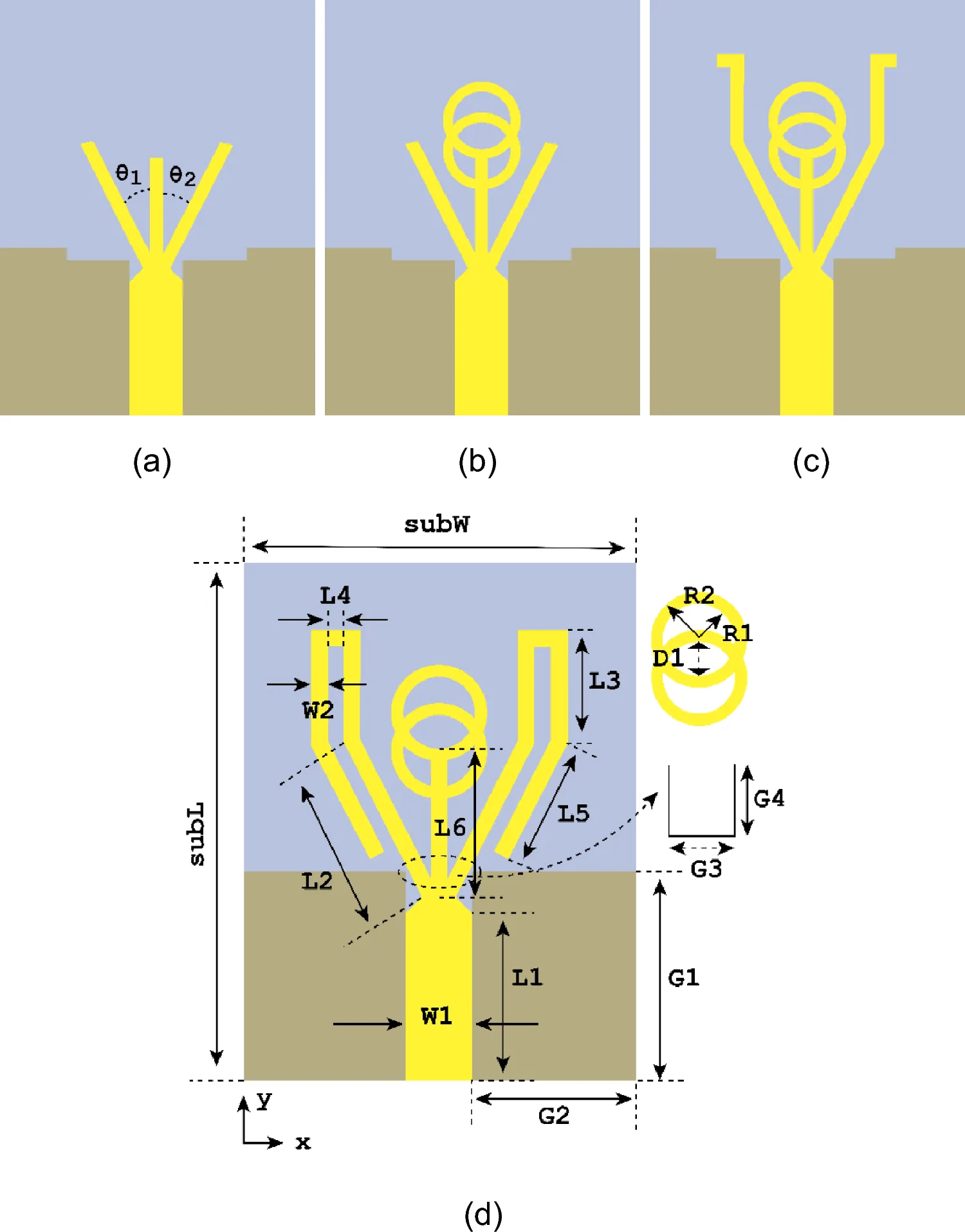
Antenna evolution stages: (**a**) stage 1, (**b**) stage 2, (**c**) stage 3, and (4) proposed single-element antenna.

The article presents a compact, wideband antenna design on a thin Rogers 5880 substrate (0.254 mm) having a dielectric permittivity $\left({\varepsilon}_{r}\right)$ of 2.2 and the loss tangent of 0.0009. The design is a monopole antenna with multiple arms and a partial ground plane. Most of the literature shows that applying partial ground as a bandwidth enhancement technique^30–32^. However, this is not the case with any monopole structure of any ground length, which could result in a wide bandwidth. Thus, the article discusses in detail the effects of partial ground on various monopole structures and their resonance properties. As a result, the ground plane length is almost kept constant at $0.3{\lambda}_{0}$ (at 28 GHz), and the monopole structure is varied in stages. The initial design (stage 1) begins with the three-arm monopole, where the left-most arm is at an angle of 27° ${(\mathrm{w}\mathrm{h}\mathrm{e}\mathrm{r}\mathrm{e} \uptheta }_{1}={\uptheta}_{2})$ from the middle arm, so as the right-most arm. The length of the left and right arms is $0.25{\lambda}_{0}$, whereas the middle arm is $0.19{\lambda}_{0}$. Further, in stage 2, the middle arm is terminated by two concentric rings. In stage 3, the left and right arms are vertically extended and terminated with a horizontal arm of length $\mathrm{L}1$. In the final stage (stage 4), the left and right arms are extended vertically downward and bent at ± 153°, as shown in Fig. 1. These evolutionary stages are achieved using CMT, which is discussed in detail in the next section.

### Characteristic mode theory (CMT)

The characteristic mode theory (CMT) is applied to the design and development of arbitrary-shaped antenna structures for which conventional theoretical expressions are difficult to evaluate. The incident electric field ${E}^{i}$ on the conductive surface of the antenna generates mode currents ${J}_{n}$, which forms the weighted orthogonal sets. The sum of these orthogonal modes determines the antenna resonance and radiation characteristics. The total current on the conductive surface is given by^17,33^,

1 $$J= \sum_{n}{\alpha}_{n}{J}_{n}$$

where the model weighted coefficient (MWC) $\left({\alpha}_{n}\right)$ is determined by considering the eigenvalue ${(\lambda }_{n})$ expression in the impedance *Z* (where *Z=R+jX)* as,

2 $$\left(R+jX\right)\left({J}_{n}\right)=(1+j{\lambda}_{n}) R\left({J}_{n}\right)$$

Solving (2) results in a modal excitation coefficient $$({V}_{n}^{i})$$, through which the MWC $${\alpha}_{n}$$ is achieved as $${\alpha}_{n}= \raisebox{1ex}{${V}_{n}$}\!\left/ \!\raisebox{-1ex}{$1+j{\lambda}_{n}$}\right.$$ that can be substituted in Eq. (1), leading to the modal current *J* as,

3 $$J= \sum_{n}\frac{{V}_{n}^{i}{ J}_{n}}{1+j{\lambda}_{n}}$$

The variation in modes, determining its characteristics as resonant or non-resonant, can be analyzed more conveniently from the inverse of the eigenvalue as,

4 $${MS}_{n}= \left|\frac{1}{1+j{\lambda}_{n}}\right|$$

On one hand, the modal significance $({MS}_{n})$ determines the mode behavior, which is normalized to 1. On the other hand, the phase angle behavior between the mode current $\left({J}_{n}\right)$ and tangential electric field ${(E}_{n})$ on the antenna geometry is determined by the characteristic angle $({CA}_{n})$^34^ that is given by,

5 $${CA}_{n}=180^\circ -{tan}^{-1}\left({\lambda}_{n}\right)$$

The eigen mode is considered to be significant if its ${MS}_{n}$ value is > 0.78 and converges to ${CA}_{n}$ angle of 180°. The mode bandwidth is determined by the steepness or shallowness of the curve at an angle of 180°, which also indicates the quality factor (Q). (i) Narrower will be the bandwidth of the mode if the slope is steep with a high Q-factor, and (ii) wider bandwidth if the slope is shallow with a low Q-factor. Furthermore, the MWC validates the mode magnitude and phase, which contribute to the total current and radiation.

#### Analysis of monopole antenna with three arms

As discussed in the earlier section, the stage 1 monopole antenna has three arms, which are at ± 27° with respect to the center arm, and the ground plane is maintained at $0.3{\lambda}_{0}$. For the CMT analysis, the substrate is assumed to be lossless, and the conductive layers are modeled as perfect electric conductors (PECs). The Integration equation solver is applied in CST Studio. The number of chosen modes is five, because for electrically small antennas, fewer modes are sufficient^17^. The overall dimension of a single-element antenna is $0.75{\lambda}_{0}\times 0.56{\lambda}_{0}$. As an evidence, the stage 1 antenna is evaluated with 10 modes and it is found that the higher order modes nither attained MS to 1 nor converge to 180°. As a result, the number of modes in subsequent evolutionary stages is limited to 5.

**Fig. 2:**
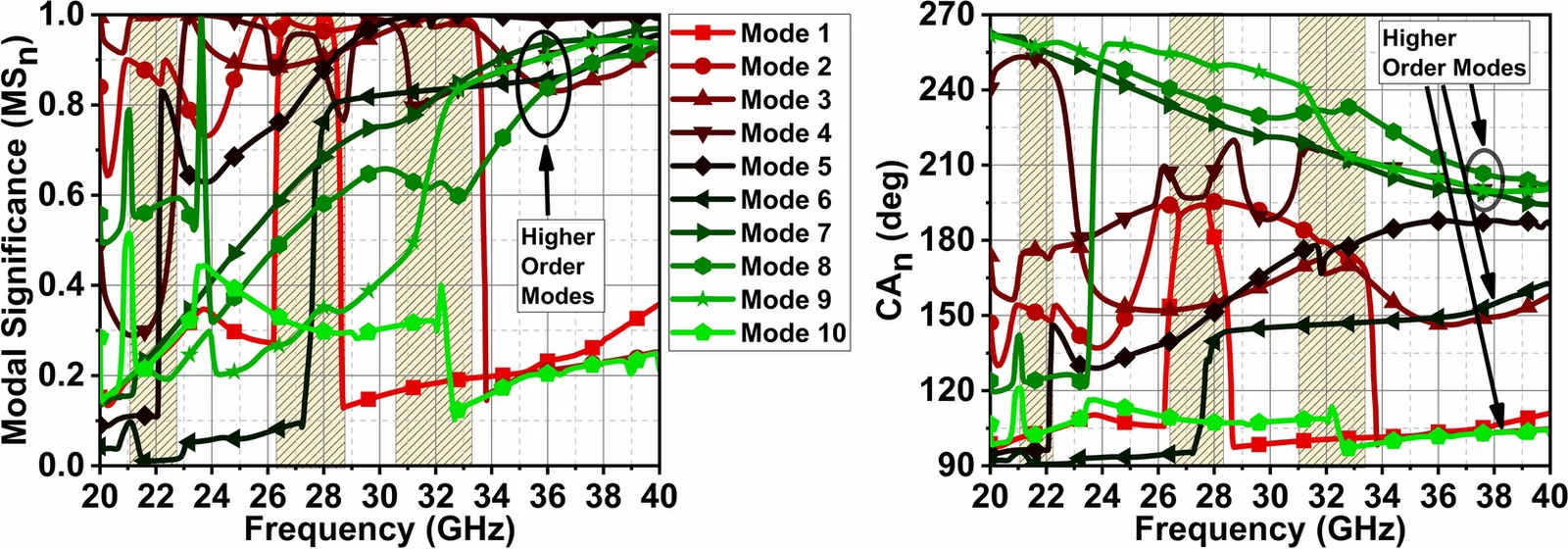
CMT results for the initial antenna, with three arms and a partial ground plane.

It is to be noted that for the initial design of a partial ground with three arms, the antenna demonstrates the narrow band operation around 21 GHz, 27.5 GHz, and 32.5 GHz with significant Modes of ${J}_{2}+{J}_{3}$, ${J}_{1}+{J}_{2}+{J}_{3}+{J}_{4}+{J}_{5}$, and ${J}_{2}+{J}_{3}+{J}_{4}+{J}_{5}$, as depicted in Fig. 2a. However, the ${CA}_{n}$ depicts the convergence at 21 GHz and 32.5 GHz due to ${J}_{3}$ and ${J}_{2}+{J}_{3}+{J}_{5}$ Modes. On the other hand, at 27.5 GHz, the convergence of ${J}_{1}+{J}_{2}+{J}_{4}$ at 190°, indicating the presence of capacitive reactance, storing the electric field in the near-field region, thereby hindering antenna performance. The Modes ${J}_{6}$ to ${J}_{10}$ attain significance only around 40 GHz, and it possesses highly capacitive and inductive reactances below this frequency range.

#### Concentric ring termination

**Fig. 3:**
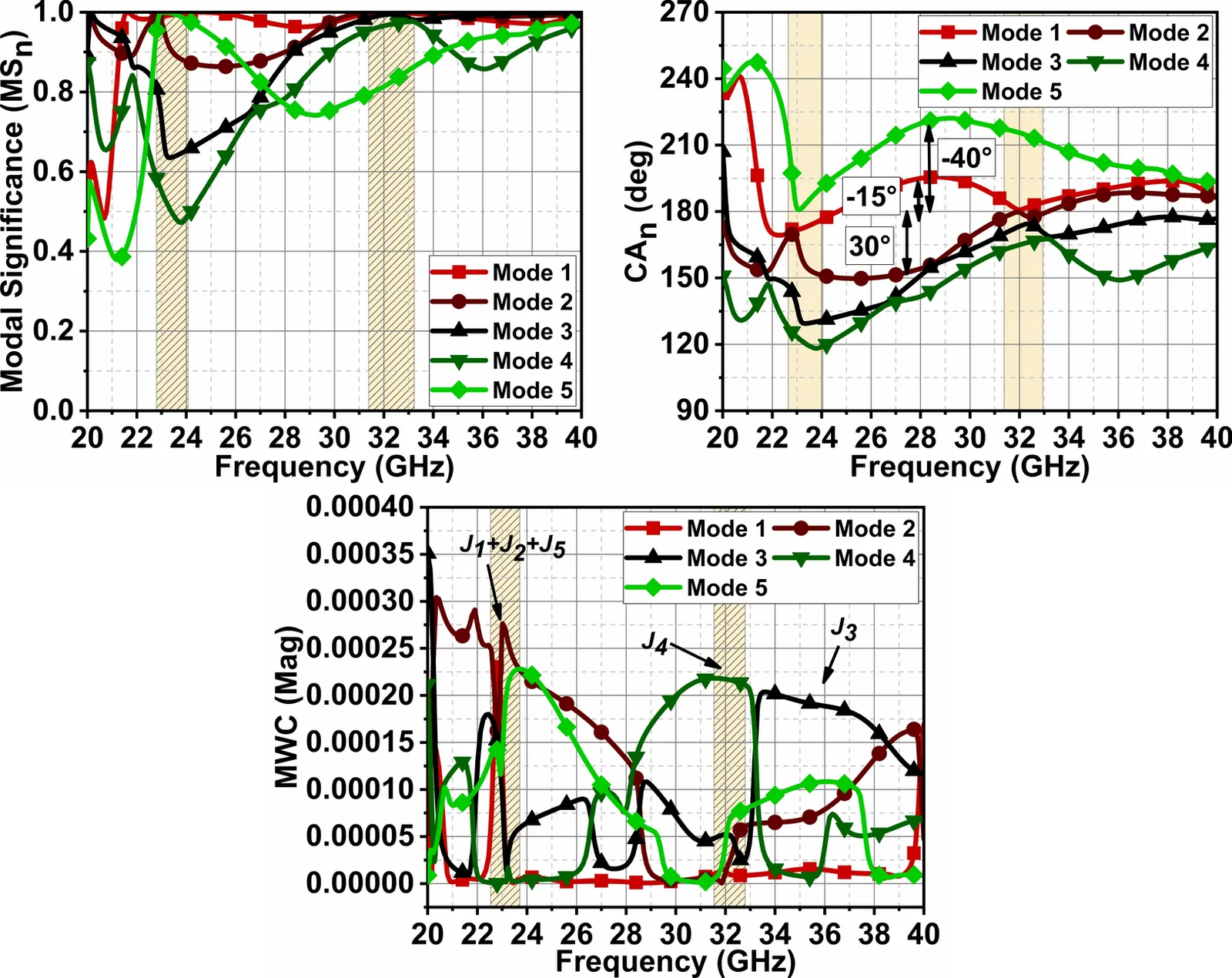
CMT results for the stage 2 antenna, with the middle arm terminated by two concentric rings.

Further, the middle arm is terminated with two concentric rings to improve higher-order modes. Figure 3 clearly illustrates that the three modes attain significance at a slightly shifted frequency of 23 GHz $({J}_{1}+{J}_{2}+{J}_{5})$, and all five modes at 32 GHz. The phase angle shows similar results, except that Mode 5 is largely capacitive, with a phase difference of − 35° at 32 GHz, and likewise, Modes 3 and 4 are inductive at 23 GHz, storing magnetic field energy in the near-field region and introducing a phase difference of approximately 60°. However, the intended resonance is at 28 GHz, where the Modes ${J}_{1}$ to ${J}_{3}$ appears to be significant, but the phase angle differences are approximately − 15°, 30°, and − 40°. The surface current interpretation predominantly strengthens the justification for the characteristic angle. The surface current of the modes ${J}_{n}$ may constitute longitudinal, transverse, or loop current. An antenna can be an electric dipole or a magnetic dipole, based on the orientation of the mode current ${J}_{n}$. For an antenna to satisfy the condition of an electric dipole, the mode currents ${J}_{n}$ has to be longitudinal or transverse. Conversely, the antenna behaves like a magnetic dipole only when the mode current ${J}_{n}$ forms the loop current, with the characteristic angle crossing at 180°. However, to achieve linear polarization, the preferred mode currents ${J}_{n}$ to possess either longitudinal or transverse current characteristics, with angle convergence at a 180°, and any loop current that attains convergence has to be suppressed.

**Fig. 4:**
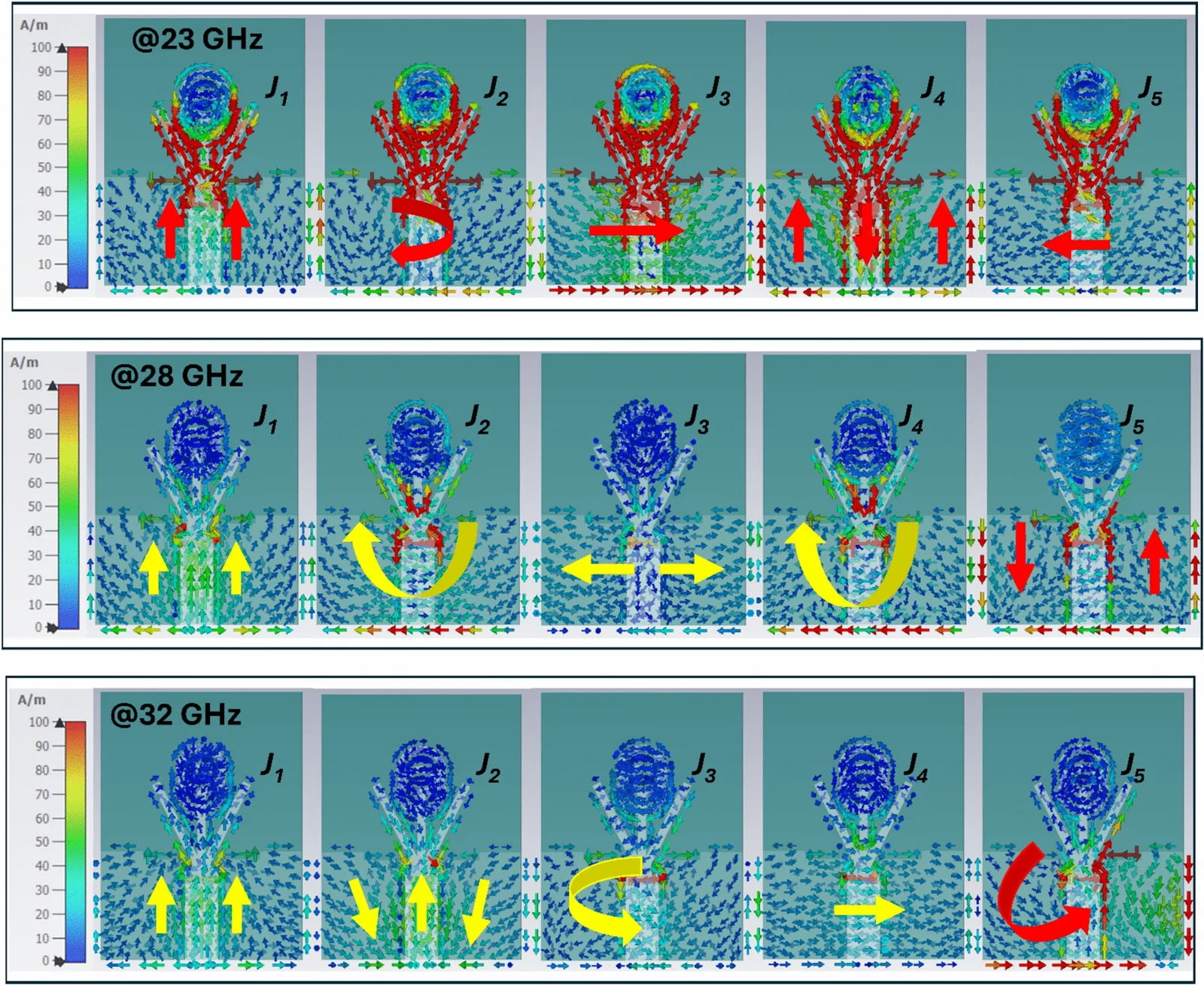
Surface current distribution results from the CMT of the stage 2 antenna.

To better understand, we analyze surface currents to characterize the behavior of electric or magnetic dipoles. Figure 4 illustrates the surface current results at 23 GHz, 28 GHz, and 32 GHz; higher current density is shown in red, and lower in yellow. It is to be observed that at 23 GHz, the converging modes are ${J}_{1}$, ${J}_{2}$, and ${J}_{5}$; the ${J}_{1}$ and ${J}_{5}$ forms the longitudinal and transverse current depicting electric dipole behavior, whereas ${J}_{2}$ creates a loop current depicting the magnetic dipole behavior. On the other hand ${J}_{3}$ and ${J}_{4}$ behaves as largely inductive modes at 23 GHz, which apparently become non-resonant despite high longitudinal and transverse currents, as verified by the MWC plot in Fig. 3. The MWC clearly depicts a higher magnitude of ${J}_{1}$, ${J}_{2}$ and ${J}_{5}$ at 23 GHz, however, the magnitude of ${J}_{2}$ drops significantly within this band, causing variation in the total current, as well as the electric dipole modes ${J}_{1}$ and ${J}_{5}$ outruns the magnetic pole behavior of ${J}_{2}$. On the other hand, the ${J}_{3}$ achieves a slightly higher magnitude just before 23 GHz and drops significantly in the band, whereas ${J}_{4}$ magnitude is low throughout. Therefore, the only modes contributing to the resonance are ${J}_{1}$+${J}_{5}$.

At 28 GHz, ${J}_{1}$ and ${J}_{2}$ shows significance > 0.8; however, these modes exhibit capacitive and inductive characteristics and do not converge. Despite their longitudinal and transverse characteristics, these modes are resonant but have low energy, as evidenced by MWC, and are unable to radiate at this frequency. At 32 GHz, multiple modes ${J}_{1}$ to ${J}_{4}$ converge at 180°; however, they carry low energy except ${J}_{3}$ and ${J}_{4}$. The ${J}_{4}$ has a higher magnitude with transverse current, whereas ${J}_{3}$ forms a loop current; as a result, the antenna exhibits electric- and magnetic-dipole characteristics and degrades impedance matching at this frequency.

#### Vertically extended arms

**Fig. 5:**
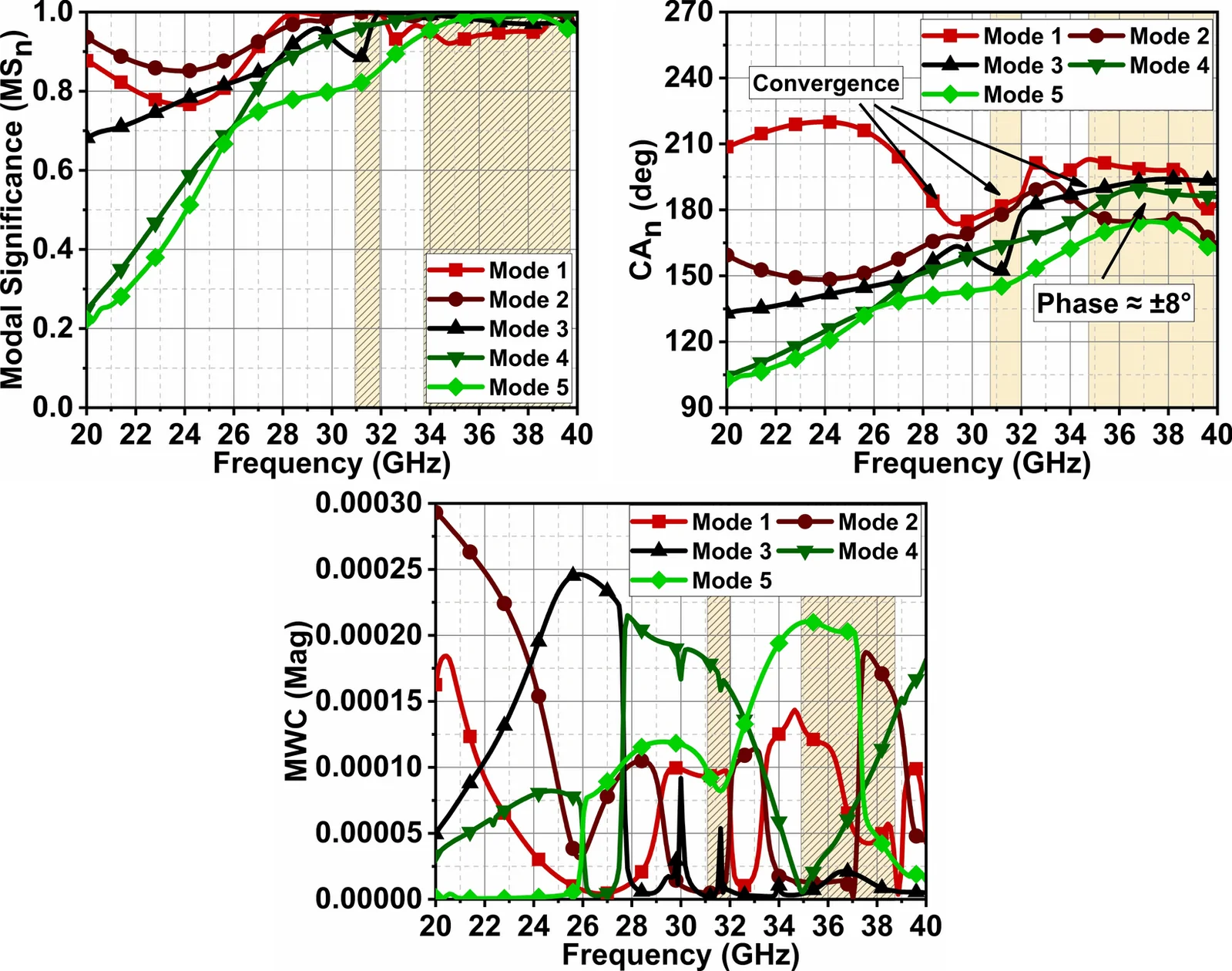
CMT results for the stage 3 antenna, with the vertically extended left and right arms.

In stage 3, the left and right arms are vertically extended by a length of $0.16{\lambda}_{0}$ and terminated by a short horizontal arm of $0.024{\lambda}_{0}$ length. The modification has significantly shifted the result from a lower resonance of 23 GHz to higher resonances at 35 GHz and above. The CMT results in Fig. 5 illustrate that the Modes ${J}_{1}$ and ${J}_{2}$ are significant and converge at 31 GHz; ${J}_{2}$ and ${J}_{4}$ at 34.5 GHz. For 34.5 GHz and above Modes ${J}_{1}$, ${J}_{3}$, and ${J}_{4}$ shows capacitive effect with an approximate phase difference of − 10°; and ${J}_{2}$, and ${J}_{5}$ with an inductive effect with a phase difference of + 8°. The phase difference of these modes is within the threshold limit of $141.26^\circ \le {CA}_{n} \le 218^\circ$ for ${MS}_{n}=0.78$. Consequently, each of these modes contributes to the antenna resonance, and opposite reactances balance its reactive components, contributing to the total current and antenna radiation.

Though ${J}_{1}$ and ${J}_{2}$ converge at 31 GHz, the surface current illustrates ${J}_{1}$ depics magnetic dipole behavior and ${J}_{2}$ electric dipole behavior and their energy levels complement each other. Contrary, the ${J}_{4}$ possessing slightly inductive characteristics and longitudinal current behavior, demonstrating a relatively higher MWC magnitude that dominates the band, and contributes to the antenna resonance, along with ${J}_{1}$. Likewise, at 34.5 GHz and above, Modes ${J}_{5}+{J}_{1}$ and ${J}_{2}+{J}_{1}+{J}_{4}$ contributes to the antenna resonance.

**Fig. 6:**
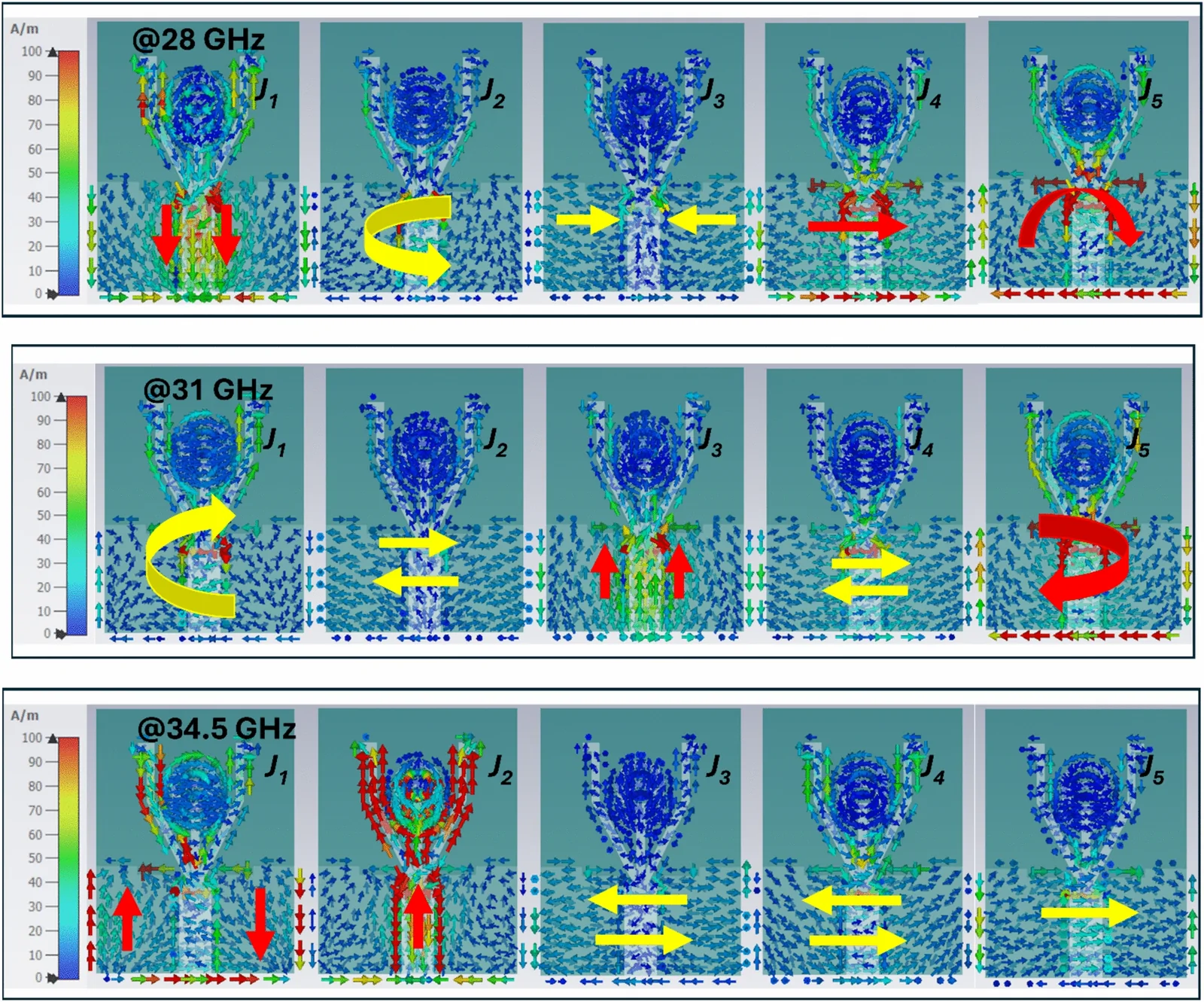
Surface current distribution results from the CMT of the stage 3 antenna.

At a lower frequency of 28 GHz, the ${J}_{1}$ is observed to cross over an angle of 180°; however, the energy level is too weak to excite the antenna. On the other hand, Modes ${J}_{3}$ to ${J}_{5}$ carry higher energy; however, due to inductive reactance, they store the magnetic energy in the near-field region and do not resonate at the given band (Fig. 6).

#### Proposed antenna structure

**Fig. 7:**
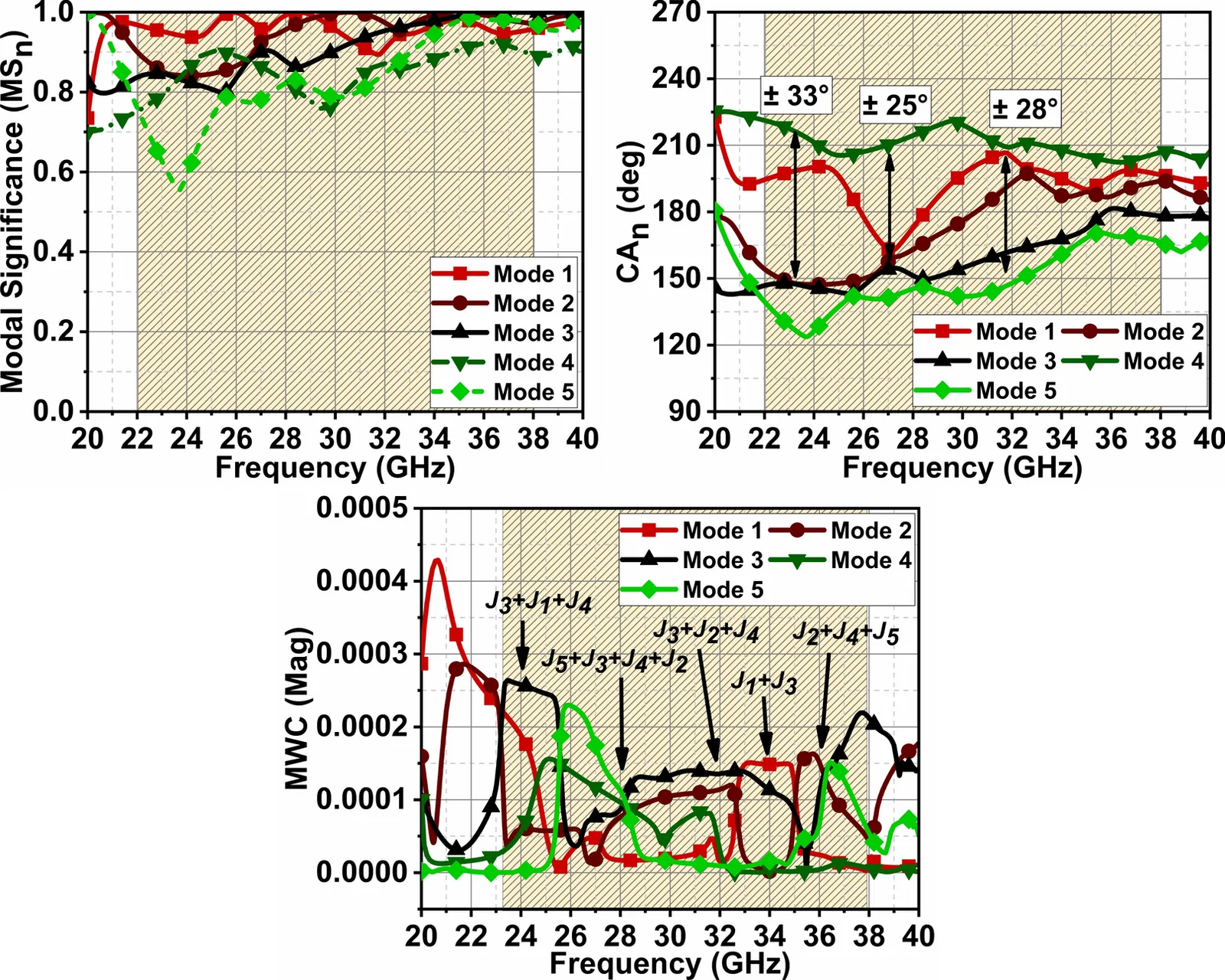
CMT results for the stage 4 (proposed) antenna, with the vertically extended down and bent.

**Fig. 8:**
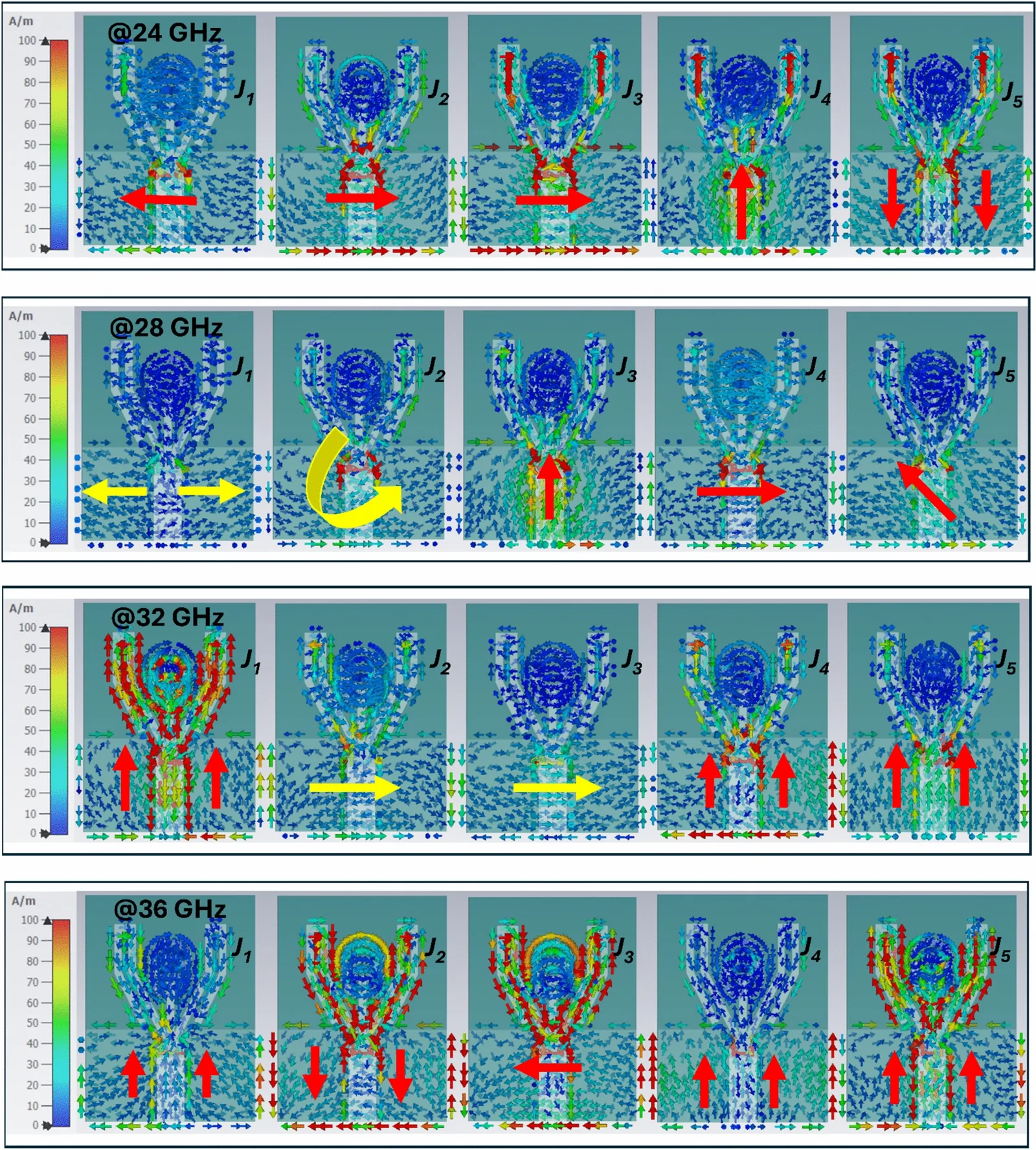
Surface current distribution results from the CMT of the stage 4 (proposed) antenna.

**Fig. 9:**
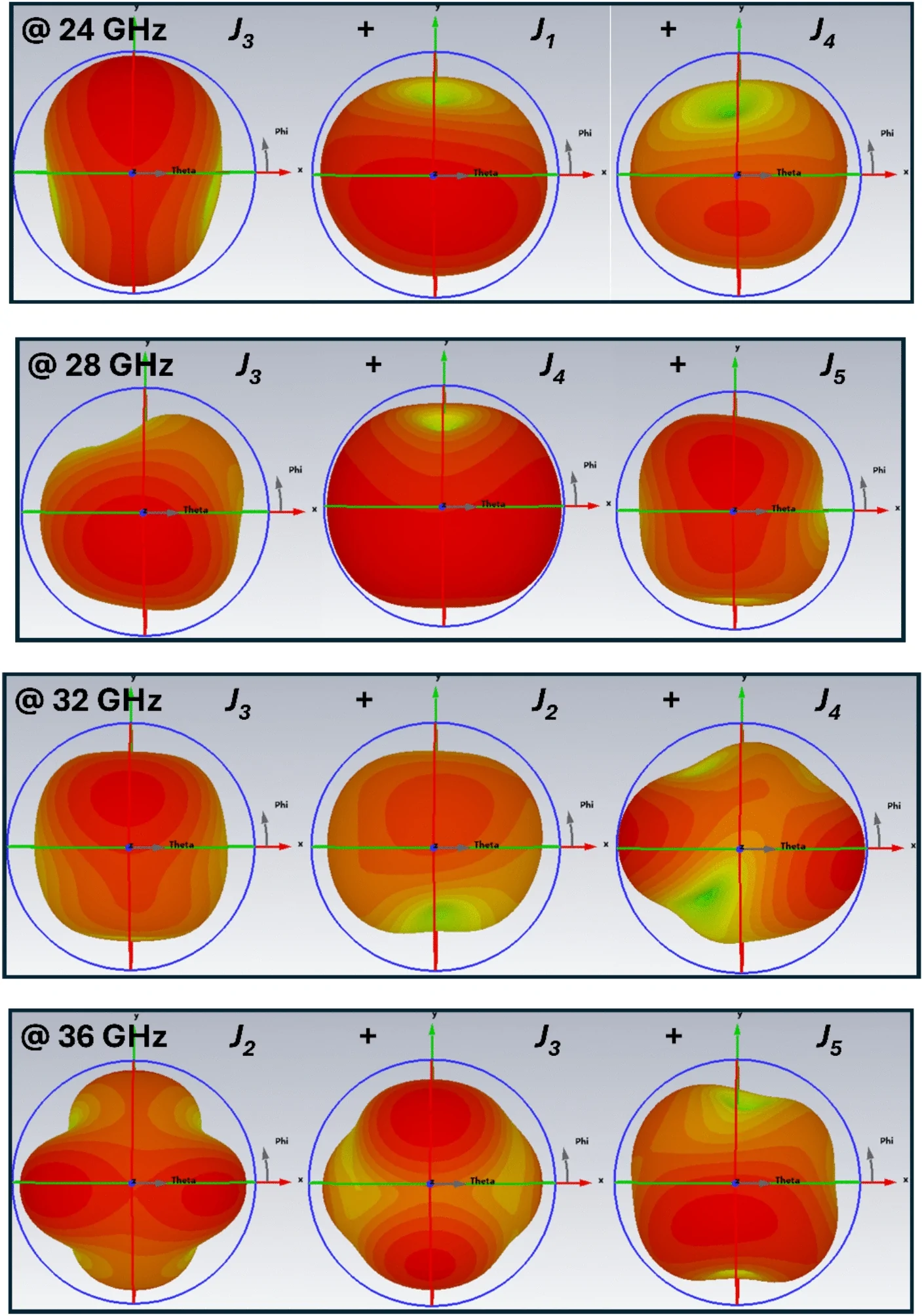
Resulting CMT radiation pattern originated from the orientation of mode currents in the proposed antenna structure.

In the final stage (stage 4), the left and right arms are further extended horizontally down by a length of $0.16{\lambda}_{0}$ and bent slightly, forming an angle of ± 153° with $0.19{\lambda}_{0}$ length. As a result, the modes are attaining significance at multiple frequency bands, contributing to the wideband resonance. At 24 GHz, ${J}_{1}$ to ${J}_{4}$ are close to the significance value of 1; however, they carry a minor capacitive (${J}_{1}$, ${J}_{4}$) and inductive (${J}_{2}$, ${J}_{3}$) reactance, as shown in Fig. 7. Nonetheless, these reactive mode angles are within the threshold limit of $141.26^\circ \le {CA}_{n} \le 218^\circ$, and they form the longitudinal/transverse current, leading to pure electric dipole characteristics (Fig. 8); as a result, a resonance at 24 GHz is expected. Observing the magnitudes of these modes, ${J}_{3}$ dominate along with ${J}_{4}$, whereas ${J}_{1}$ decreases with the increase in frequency in this band; and ${J}_{2}$ carry relatively low energy and do not contribute to the resonance. Therefore, at 24 GHz, the modes contributing to resonance are ${J}_{3}+{J}_{1}+{J}_{4}$. The in-phase and anti-phase currents of the longitudinal/transverse modes generate the radiation pattern, and their sum yields the overall radiation pattern. The ${J}_{3}$ resulted in a transverse mode with the in-phase current along the top and bottom horizontal edges of the antenna, which results in a constructive radiation pattern along the horizontal edges. In contrast, the vertical edges have anti-phases current (opposite directions), which nullifies the currents and results in a radiation null in Fig. 9. Similarly, the patterns are formed for the other modes as well, and the summations of all three modes result in an overall pattern at 24 GHz, and this follows for all the bands.

At 28 GHz, ${J}_{1}$ is observed to be converging at 180° with a sharp slope, depicting a narrow bandwidth, whereas, ${J}_{2}$, ${J}_{3}$ and ${J}_{5}$ possesses inductive reactance and ${J}_{4}$ possesses capacitive reactance, however, within the limits of ${CA}_{n}$ with mostly electric dipole characteristics, except ${J}_{2}$. The energy level of these modes is almost the same, except for ${J}_{1}$, therefore, the modes contributing to resonance are ${J}_{3}+{J}_{4}+{J}_{5}$, which dominates the ${J}_{2}$ (magnetic dipole), leading to an electric dipole behavior.

Further at 32 GHz, ${J}_{2}$ illustrate a wideband nature with a shallow slope crossover at 180° along with ${J}_{1}$, ${J}_{4}$ (capacitive modes) and ${J}_{3}$ (inductive mode). All these modes depict electric dipole behavior with ${J}_{3}$ being in the dominant mode with a higher energy level, followed by ${J}_{2}$ and ${J}_{4}$. As these three modes contribute to resonance, their reactances are nullified. At 36 GHz, all five modes are significant and closely converge at an angle of 180°, with the modes exhibiting electric-dipole characteristics. Out of these, Modes ${J}_{2}$, ${J}_{3}$ and ${J}_{5}$ are dominant due to their relatively higher energy levels. Therefore, out of five, only three modes are contributing to the resonance at 36 GHz (${J}_{2}+{J}_{3}+{J}_{5}$). Therefore, the proposed antenna excites multiple modes are various bands to achieve wideband characteristics that is ${J}_{3}+{J}_{1}+{J}_{4}$ at 24 GHz, ${J}_{3}+{J}_{4}+{J}_{5}$ at 28 GHz, ${J}_{3}+{J}_{2}+{J}_{4}$ at 32 GHz and ${J}_{2}+{J}_{3}+{J}_{5}$ at 36 GHz.

### Monopole with excitation

**Fig. 10:**
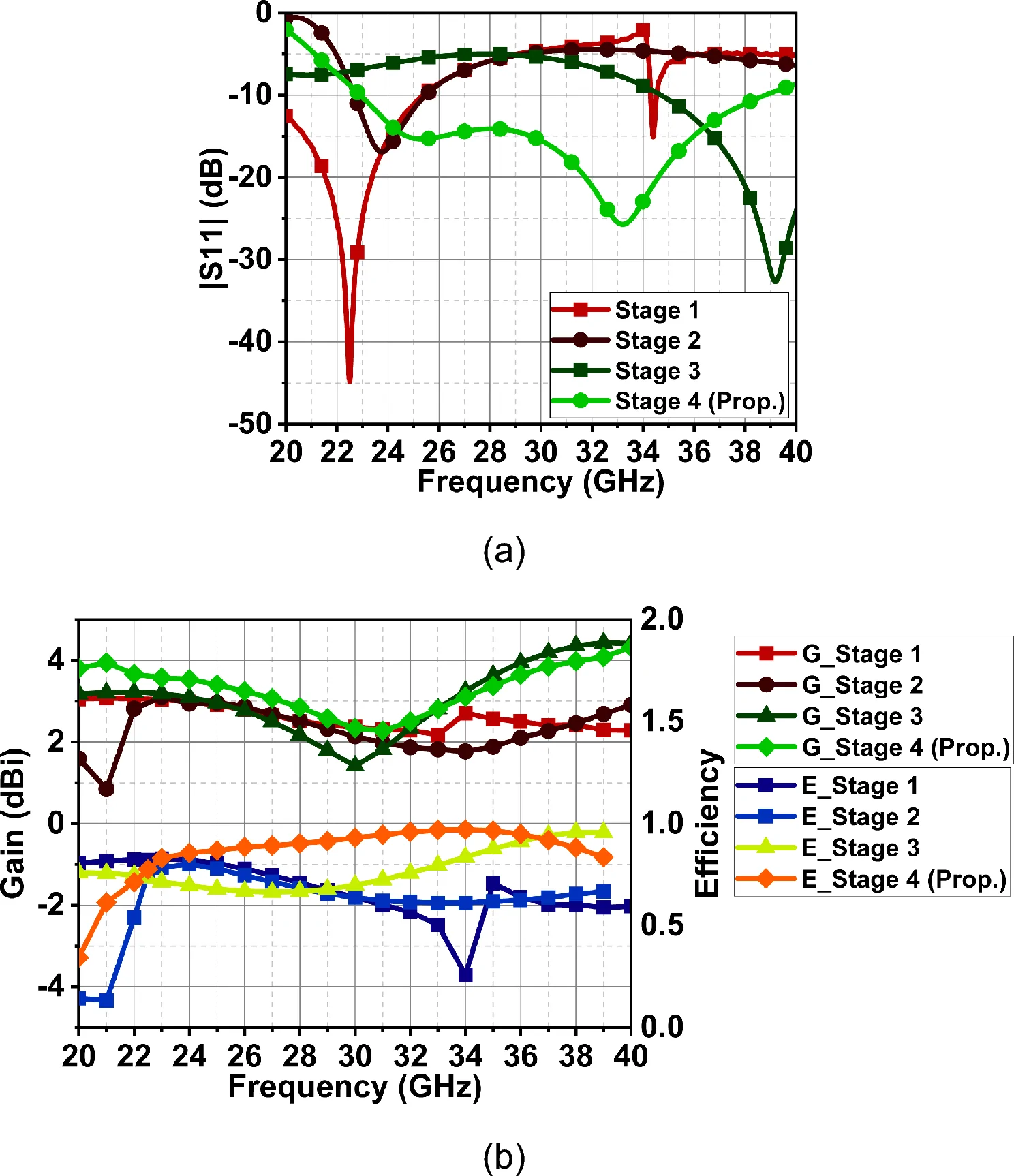
Results of antenna stages for port excitation. (**a**) Reflection coefficient |S11|, and (**b**) gain with efficiency plot.

In this section, all the antenna evolution stages are verified with their respective port excitations. In stage 1, with just three arms at 27° apart, whose left and right arm lengths are $0.25{\lambda}_{0}$ and the middle arm length of $0.19{\lambda}_{0}$ with the fixed ground length of $0.3{\lambda}_{0}$. It achieved good impedance matching |S11| of 45 dB at 22.5 GHz, with a bandwidth of 20–25 GHz, and a second band at 34.3 GHz, as shown in Fig. 10a. These results are closely correlated with the CMT results, which demonstrated two bands at 21 GHz and 32.5 GHz. In the next stage, the middle arm is terminated with two concentric rings that improve the model significance and convergence at 23 GHz and 32 GHz. However, the only electric dipole modes that dominated are ${J}_{1}$ and ${J}_{5}$ at 23 GHz, which closely matched the port excitation results at 24 GHz, with slightly reduced impedance matching. At 32 GHz, as the ${J}_{4}$ and ${J}_{3}$ carried electric and magnetic dipole characteristics; due to the orthogonal behavior of the modes, this leads to impedance mismatch in the antenna. Further, with fixed ground length and extended arm lengths, the resonance is expected to shift to lower frequencies; however, the results are the opposite. The resonance is shifted to a higher frequency, as shown by the CMT and excitation results, which occur at 39 GHz. In the final stage, the capacitive effect is increased to counter the inductive effect by extending the arms downward, thereby activating multiple modes and achieving significance over a wide frequency range. As a result, a wide bandwidth of 23–38 GHz is achieved. The proposed antenna has achieved higher gain and efficiency than earlier stages, as illustrated in Fig. 10b. However, 5G applications require high-gain antennas to compensate for the high propagation losses at mmWave frequencies. Consequently, the proposed antenna is expanded to an eight-element linear array and discussed in the next section.

### Array antenna

**Fig. 11:**
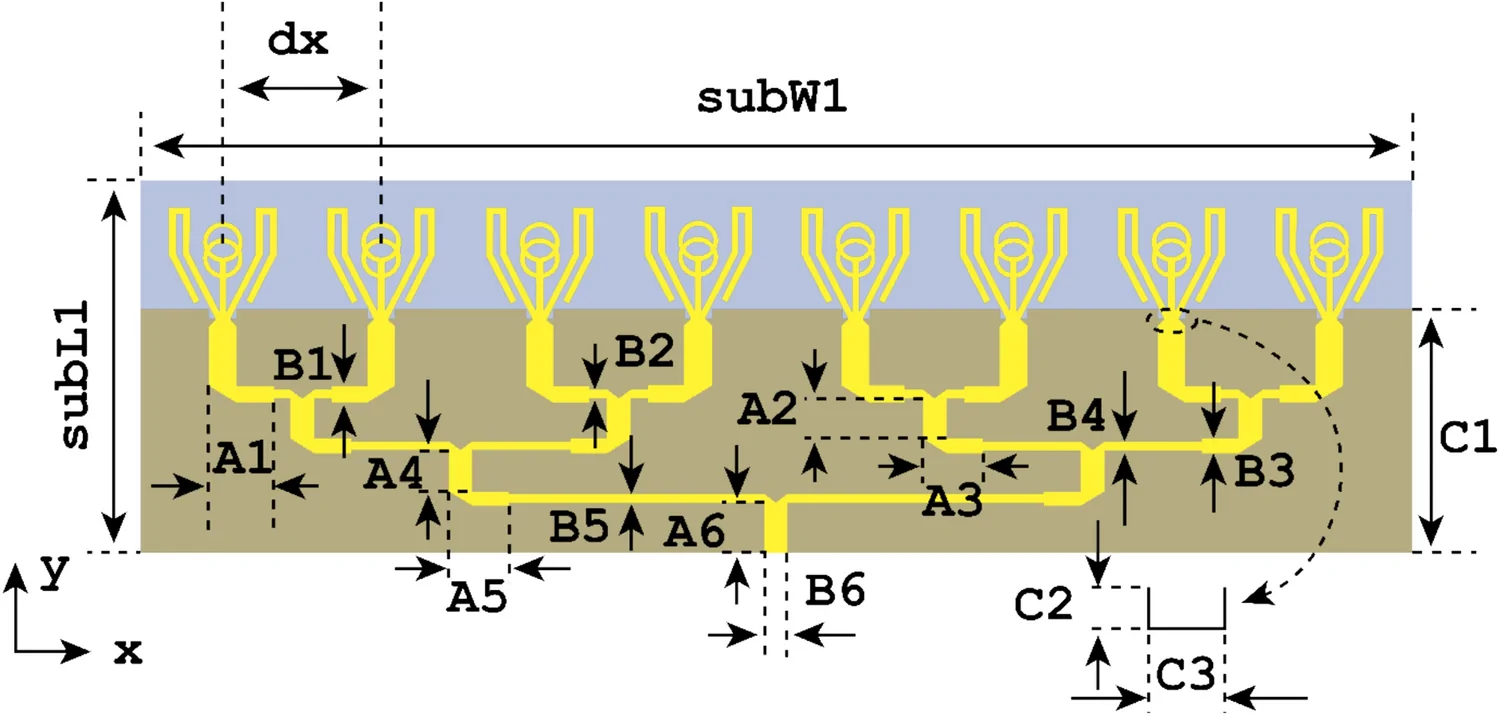
Proposed eight-element linear array antenna.

The single-element antenna shown above is expanded to an eight-element linear array to achieve high gain and a narrow beam with low side lobe levels (SSL). However, two challenges are (i) mutual coupling reduction between the antenna elements and (ii) design of the feed network for wideband operation. The first issue is addressed by the adequate spacing between the antenna elements with a distance of approximately half-wavelength, that is $\mathrm{d}\mathrm{x}=0.56{\lambda}_{0}$ and the antenna structure itself, due to the bent arms, the coupling is reduced. The second issue is addressed by designing a three-layer, T-junction feed network optimized in stages. The proposed array antenna is shown in Fig. 11.

**Fig. 12:**
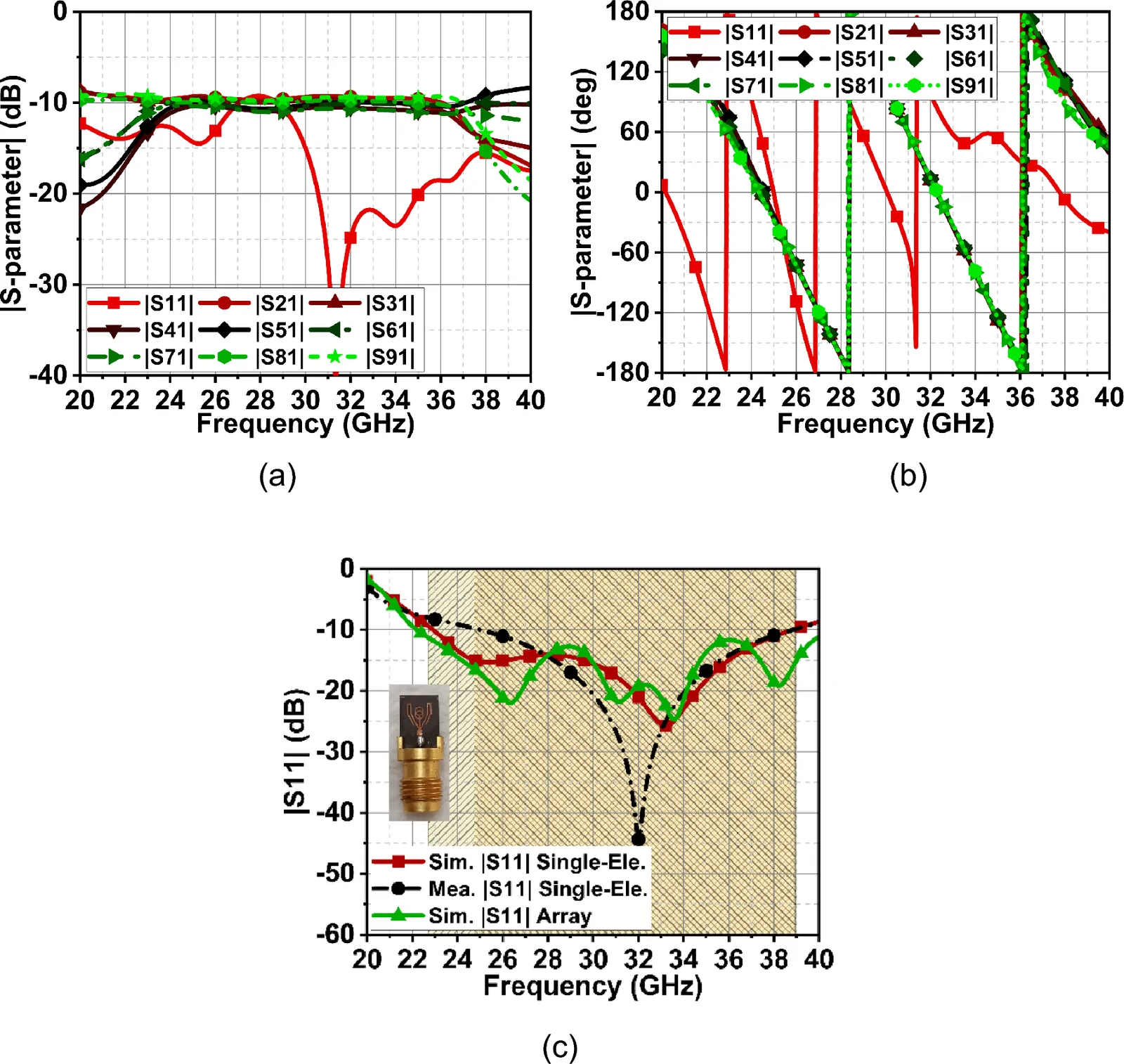
(**a**,**b**) Feed network power and phase distribution of the proposed array antenna. (**c**) Reflection coefficient |S11| results of the proposed array antenna compared with the single-element antenna.

Designing a wideband feed network is challenging because a designed network may deliver equal power and phase only within a certain frequency band, and, as frequency increases, performance may degrade. So the simultaneous design of a three-layer feed network becomes challenging. In our case, two approaches are followed: (i) a layer-by-layer design of the feed network and (ii) Final optimization of the ground plane to improve the impedance matching at the desired frequency and achieve a wide bandwidth. Initially, a two-element linear array is designed, with the first layer forming a T-junction. Here, a 50 Ω $({Z}_{A2})$ feed line delivers power to two 50 Ω antenna elements via a 100 Ω line ${(Z}_{B2})$. The impedance of ${Z}_{B2}$ to match with ${Z}_{A2}$, where ${Z}_{B2}$ forms a parallel network to a series ${Z}_{A2}$, consequently, the impedance $\left({Z}_{B2}\bullet {Z}_{B2}\right)/\left({Z}_{B2}+{Z}_{B2}\right)$ = 50 Ω matches the impedance of ${Z}_{A2}$. Further, to match the impedance of a 50 Ω antenna element with ${Z}_{B2}$, a quarter-wave-length transmission line of 70.7 Ω $\left(\sqrt{{Z}_{antenna}\times {Z}_{B2}}\right)$ impedance is used. To achieve a wide bandwidth response, the length of the quarter-wave transmission line must be tuned to match the desired bandwidth; therefore, for our case, the best response is achieved for $0.22{\lambda}_{0}$. A similar process is applied to the next two layers of the feed network; the final power distribution and phase response are shown in Fig. 12a,b. With these settings, the array antenna initially achieved the wide dual-band resonance at 23.5 GHz (21.22–26.79 GHz) and 35.5 GHz (30.3–5–36.64 GHz). Furthermore, the ground notch beneath the antenna element feed line is reduced to $0.25{\lambda}_{0}$, after which a wideband resonance is obtained. The final tuned parameters are listed in Table 1, and the achieved antenna bandwidth ranges from 22.2 to 40 GHz, as shown in Fig. 12c. The reflection coefficient |S11| of the array antenna is compared with the results of the single-element antenna to observe the discrepancies caused by mutual coupling. The simulated results indicate that the array antenna's performance is almost identical to that of a single element, indicating that the effect of mutual coupling is minimal due to the approximate half-wavelength spacing and the antenna structure itself. However, a minor discrepancy is observed in the measured results of the single-element antenna, which resulted in good impedance matching of 45 dB at 32 GHz with a slightly reduced bandwidth (at lower frequencies). The reasons for the measured discrepancies are discussed in the results section.

**Table 1:** Dimensions of antenna in mm.

Parameter	Value	Parameter	Value	Parameter	Value
$\mathrm{L}1$	2.6	$\mathrm{L}2$	2.67	$\mathrm{L}3$	1.75
$\mathrm{L}4$	0.25	$\mathrm{L}5$	2.00	$\mathrm{L}6$	2.03
$\mathrm{R}1$	0.35	$\mathrm{R}2$	0.65	$\mathrm{D}1$	0.35
$\mathrm{W}1$	0.98	$\mathrm{W}2$	0.25	$\mathrm{G}1$	3.25
$\mathrm{G}2$	2.50	$\mathrm{G}3$	1.00	$\mathrm{G}4$	1.00
$\mathrm{s}\mathrm{u}\mathrm{b}\mathrm{L}$	8.00	$\mathrm{s}\mathrm{u}\mathrm{b}\mathrm{W}$	6.00	$\mathrm{s}\mathrm{u}\mathrm{b}\mathrm{L}1$	14.00
$\mathrm{s}\mathrm{u}\mathrm{b}\mathrm{W}1$	48.00	$\mathrm{A}1$	2.40	$\mathrm{A}2$	1.69
$\mathrm{A}3$	2.20	$\mathrm{A}4$	1.68	$\mathrm{A}5$	2.2
$\mathrm{A}6$	1.9	$\mathrm{B}1$	0.45	$\mathrm{B}2$	0.20
$\mathrm{B}3$	0.42	$\mathrm{B}4$	0.22	$\mathrm{B}5$	0.20
$\mathrm{B}6$	0.78	$\mathrm{C}1$	9.15	$\mathrm{C}2$	0.25
$\mathrm{C}3$	1.00				

The expression of the array factor for the configuration is given as,

6 $$\mathrm{A}\mathrm{F}= \sum_{n=0}^{N-1}{e}^{jn\varphi }$$

where,

7 $$\varphi =kdx\mathit{sin}\left(\theta \right)+\beta $$

For an antenna element fed with equal phase, the progressive phase shift *(β )* will be zero. Thus, the normalized array factor (for *N* = 8 and $\mathrm{d}\mathrm{x}=0.56{\lambda}_{0}$, *φ * reduced to $1.12\pi \bullet \mathrm{s}\mathrm{i}\mathrm{n}(\theta )$) and power pattern are given as,

8 $${\mathrm{A}\mathrm{F}}_{n}\left(\theta \right)= \frac{1}{8}\frac{\mathrm{s}\mathrm{i}\mathrm{n}(4.48\pi \bullet \mathrm{sin}\left(\theta \right))}{\mathrm{s}\mathrm{i}\mathrm{n}(0.56\pi \bullet \mathrm{sin}\left(\theta \right))}$$

9 $$\mathrm{P}\left(\theta \right)={\left|{\mathrm{A}\mathrm{F}}_{n}\left(\theta \right)\right|}^{2}$$

**Fig. 13:**
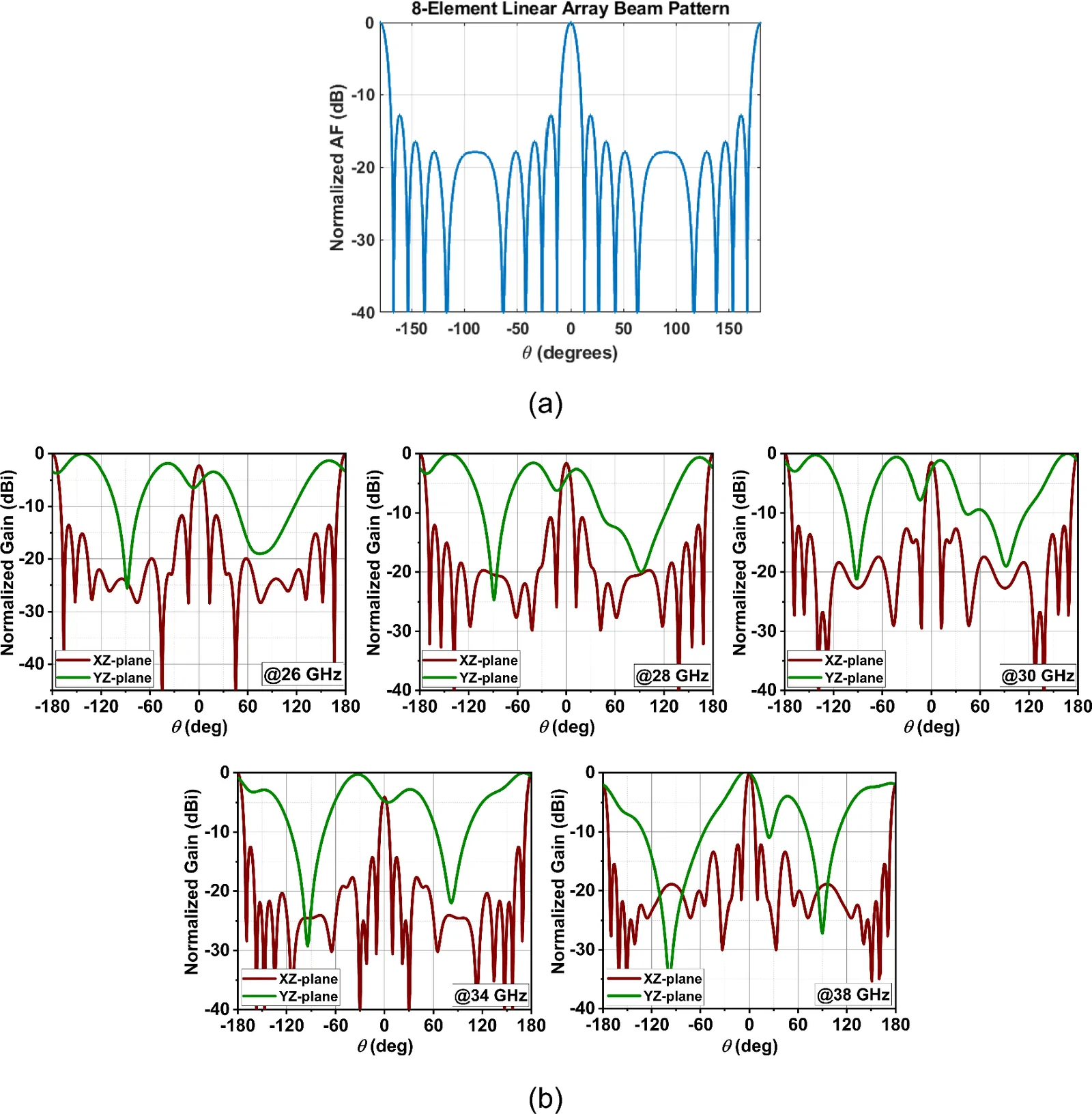
(**a**) Estimated and (**b**) simulated radiation of the array antenna.

**Fig. 14:**
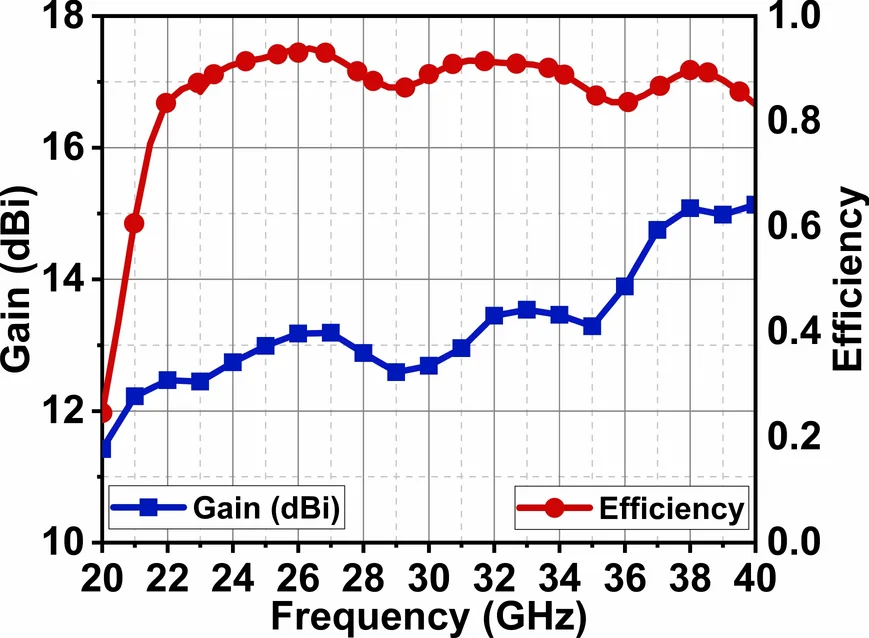
Simulated gain and efficiency plot of the proposed array antenna.

Thus, the estimated and simulated patterns are shown in Fig. 13. The estimated and simulated results are highly correlated with the half-power beamwidth (HPBW) of 12° in the XZ-plane and a wide beam in the YZ-plane. The antenna achieved a consistent narrow beam pattern across the wide bandwidth, with decent SLL ranging from − 10 dB to − 11 dB. It has also achieved high gain, with frequency-dependent performance ranging from 13 to 15 dBi, and small marginal variation in efficiency at 90%, as illustrated in Fig. 14.

## Results and discussion

**Fig. 15:**
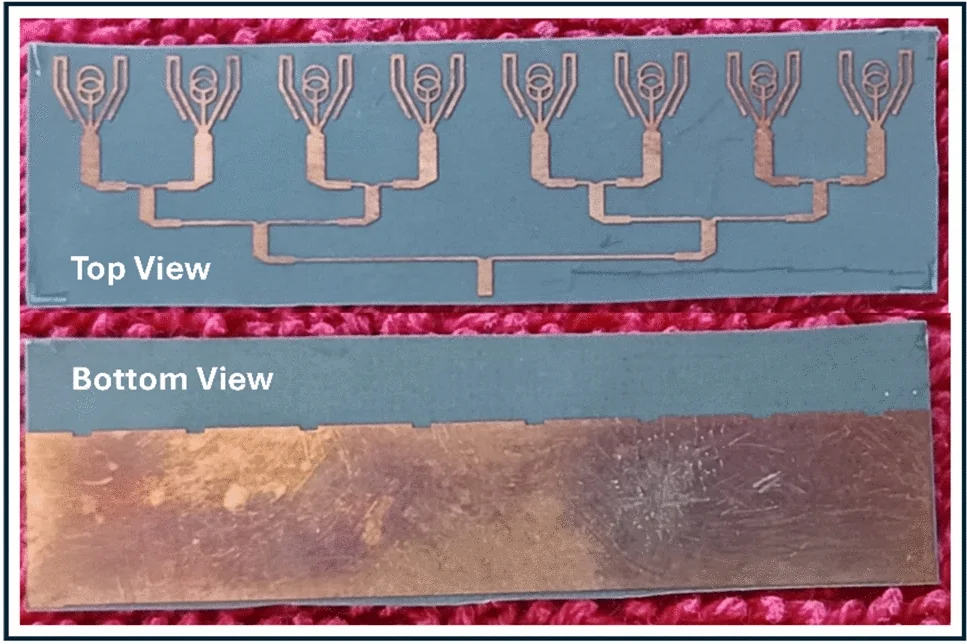
Prototype fabricated array antenna.

The single-element antenna is evolved through the CMT process for wideband operation, which is extended to an 8-element array antenna with an inter-element spacing of approximately $0.5{\lambda}_{0}$ at 28 GHz. The antenna achieved a narrow beam with decent SLL. To validate the simulated results, a prototype antenna is fabricated, as shown in Fig. 15. A 50 Ω SMA connector with an operating frequency up to 50 GHz is used to interface the antenna with a Vector Network Analyzer (VNA). Several challenges arise during the fabrication and measurement of mmWave antennas, including fabrication tolerances, port interfaces, alignment of the reference antenna (Ref-ANT) and antenna-under-test (AUT), cable losses, and cable phase shifts. However, utmost care is taken during fabrication, using a 4-mill precision milling machine, and the VNA is calibrated before measurement with a calibration kit (which includes open, short, load, and through configurations). To measure the S-parameter and radiation pattern, the two-port VNA (S820E) is calibrated from 20 to 40 GHz frequency range using a calibration kit. Furthermore, the reflection coefficient |S11| is measured using port 1 of the VNA, and the two-port approach is used to measure the radiation pattern, with the reference antenna (Ref-ANT) connected to port 1 and the antenna under test (AUT) connected to port 2. The radiation pattern is measured in an anechoic chamber, with the reference antenna operating over 18–40 GHz, and the test antenna is the proposed array structure. The VNA sends power from port-1 to the reference antenna, which transmits the signal, and the AUT receives the signal and couples it to port-2.

**Fig. 16:**
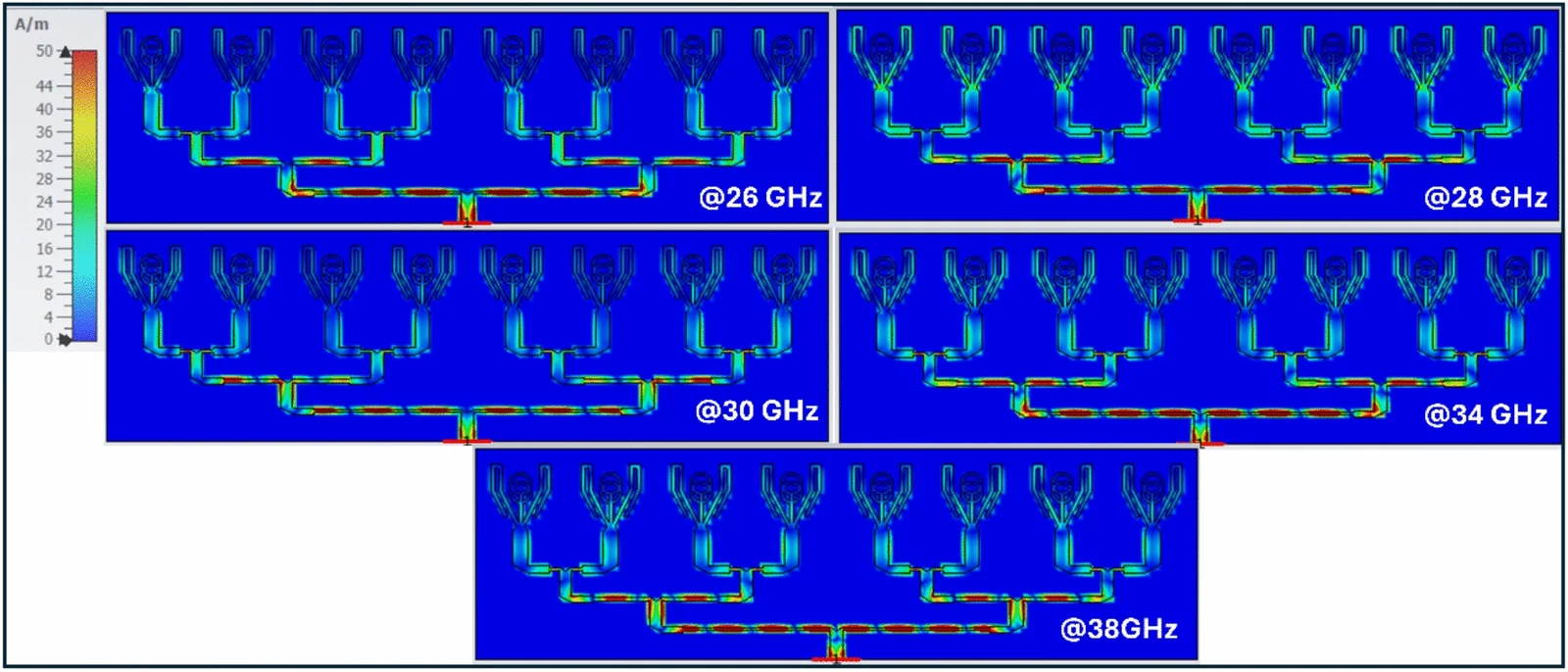
Illustrating an equal surface current distribution to all the elements across the bandwidth.

**Fig. 17:**
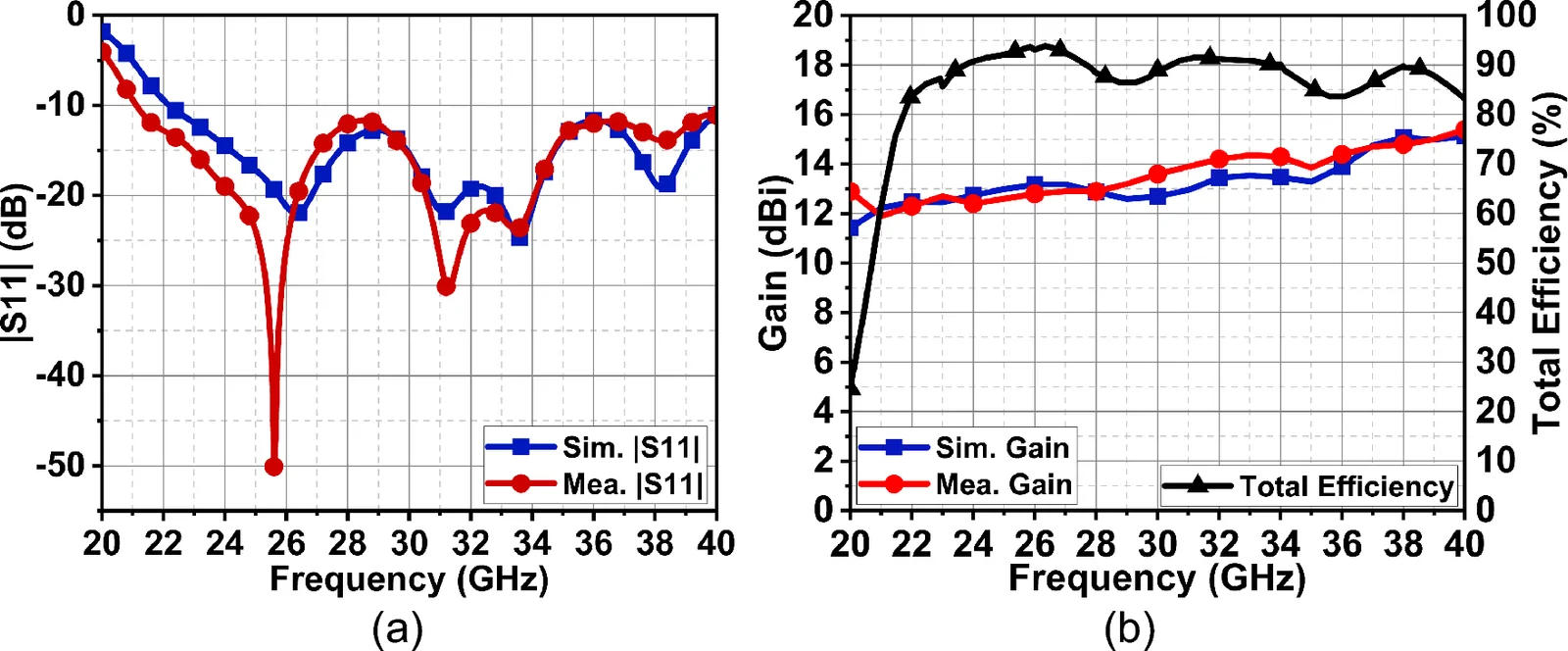
Simulated and measured (**a**) reflection coefficient |S11| and (**b**) gain and efficiency.

**Fig. 18:**
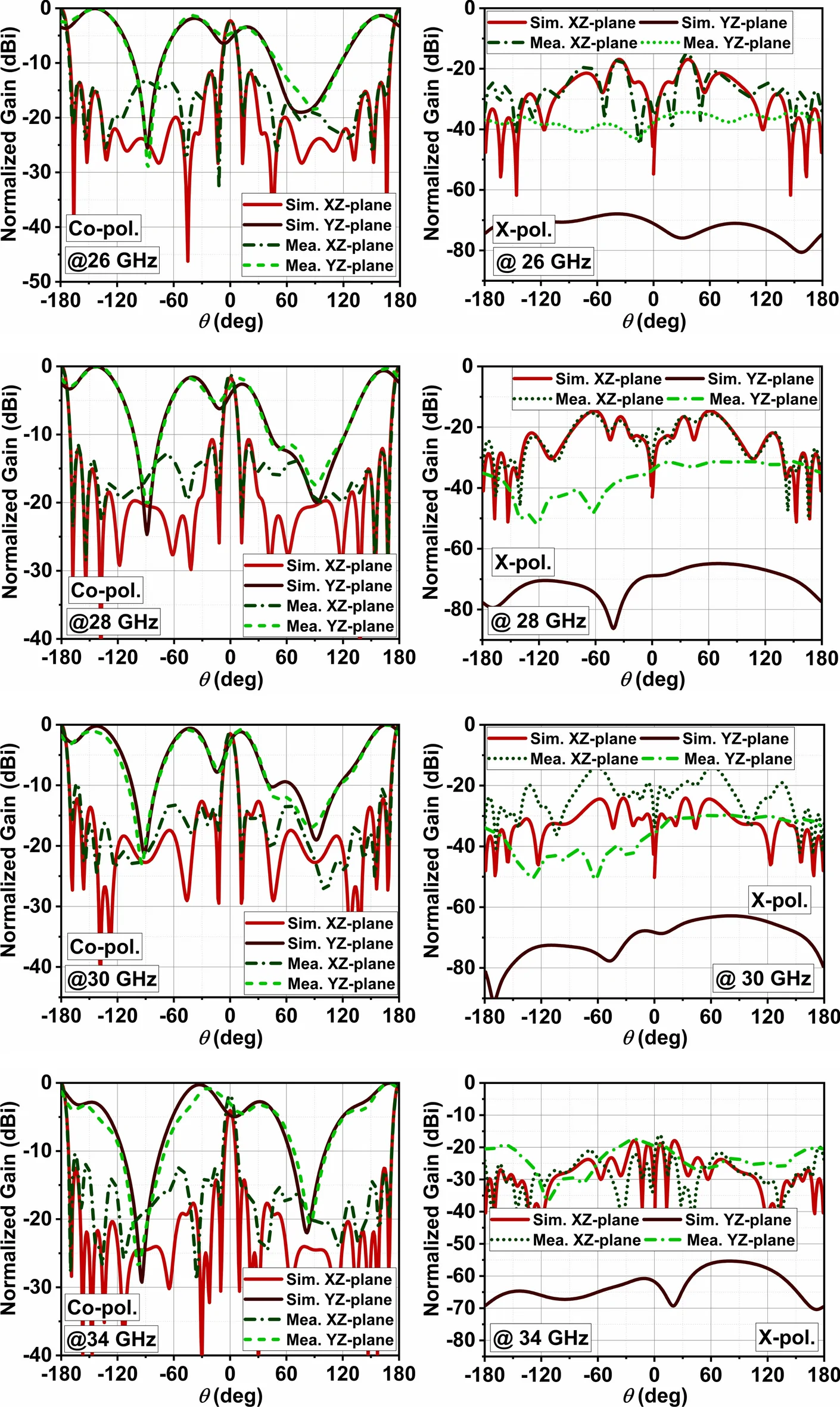

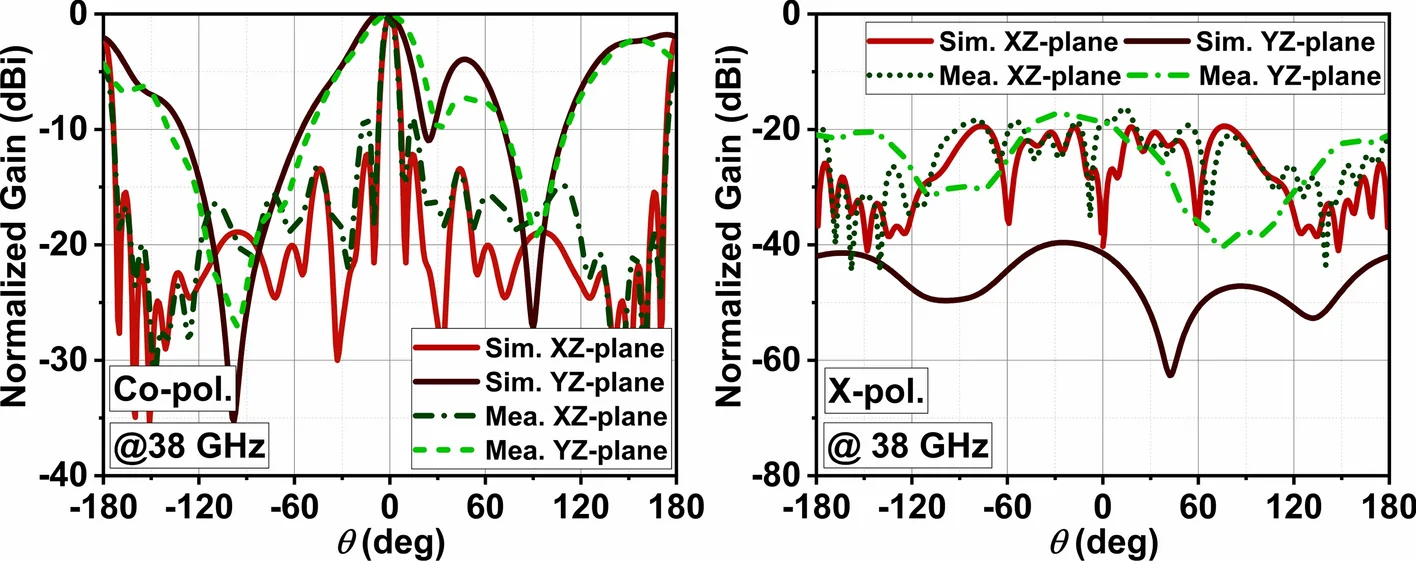
Simulated and measured radiation pattern of the proposed array antenna.

The array antenna used a three-T-junction feed network, and the ground slits were optimized to match the impedance over a wide bandwidth. Figure 16 illustrates the equal current distribution across the bandwidth depicted at 26 GHz, 28 GHz, 30 GHz, 34 GHz, and 38 GHz. The simulated and measured reflection coefficients |S11| show good agreement in Fig. 17, resulting in bandwidths of 22.2–40 GHz and 21.1–40 GHz, respectively. Also, the measured results show high impedance matching at 25.6 GHz, 31.1 GHz, and 33.3 GHz. The antenna simulated and measured HPBWs in the XZ-plane are 12° and 10°, 10.5° and 10°, 11° and 10°, 09° and 09°, and 09° and 09° at 26 GHz, 28 GHz, 30 GHz, 34 GHz, and 38 GHz, respectively. The designed structure has resulted in a decent SLL of − 10 to − 11 dB for both simulated and measured results, as shown in Fig. 18. The antenna has also demonstrated good cross-polarization of < − 18 dB (simulated) and < − 17.5 dB (measured) in the XZ-plane and < − 55 dB (simulated) and < − 30 dB (measured) in the YZ-plane, across the bandwidth. The simulated and measured gains increase almost linearly from 12 dBi to 15.5 dBi, as depicted in Fig. 17b.

Table 2 presents a detailed comparison between the proposed antenna and recently reported mmWave antenna arrays. The comparison highlights that several existing designs achieve high gain by using complex structures such as stacked substrates, SIW cavities, metasurface layers, superstrates, reflectors, or special endfire feeding arrangements. In contrast, the proposed work provides a wide operating bandwidth of 22.5–39 GHz, a compact single-element size, and a 1 × 8 feed-array configuration. The proposed antenna covers both the 28 GHz and 38 GHz 5G mmWave bands, whereas many reported works are primarily limited to the 24/28 GHz region. Moreover, the proposed array achieves a high-gain range of 11–15.8 dBi and a radiation efficiency greater than 85%. A key distinction of this work is the use of characteristic mode analysis, which explains the antenna's modal contributions and radiation mechanism rather than relying solely on conventional geometric optimization. Therefore, Table 2 compares the proposed antenna with the reported designs; it offers a balanced combination of compact size, wide bandwidth, high gain, good efficiency, low cross-polarization, and a physically interpretable CMT-based design.

**Table 2:** Comparison table of the proposed antenna with published literature.

Ref	Dim in λ^3^	Array Size L × W (mm^2^)	Res. GHz	Design Technique	Array Conf	BW in GHz	Gain (dBi)	Avg. Total/ Rad* Effi. (%)
^18^	4.20 × 1.87 × 0.024	45 × 20	28	Wire-hexagon planar array with 4-way feed network	1 × 4	25.052–34.923	12.15	> 85*
^19^	3.51 × 1.33 × 0.024	37.6 × 14.3	28	Modified bowtie planar array with corporate feed network	1 × 4	23.41–33.92	10.7	> 90*
^20^	2.99 × 1.12 × 0.024	32 × 12	28	Snowflake fractal antenna array with corporate feed network	1 × 4	27.45–28.86	10.15	> 90*
^22^	0.025	NAV	30	Compact asymmetric dipole array with leaky-wave coupling behavior	1 × 8	25–35	> 7	NAV
^23^	2.07 × 2.08 × 0.051	20.5 × 20.6	30	Metasurface-based Fabry–Perot cavity antenna array	1 × 2	27.38–33.34	12.7	92
^24^	7.18 × 5.69 × NAV	77 × 61	28	X-shaped slot array with reflector and dual superstrate layers	4 × 4	25.35–25.95, 26.8–30.2	17.5–20	NAV
^25^	5.00 × 2.30 × NAV	61.2 × 28.2	24.5	Colocated dual-mode circular patch array for SLL reduction	1 × 4	23.6–24.3	7.2	NAV
^26^	3.41 × 4.76 × 0.912	34.1 × 47.6	28	Stripline-fed radiating-via monopole endfire array	1 × 8	24–30.12	15.76	91.22*
Prop	1.31 × 4.48 × 0.024	14 × 48	28/38	CMT-guided wideband mmWave antenna with feed-array configuration	1 × 8	22.5–39	15.8	> 85

Ref	Beam Width (deg)		X-polarization (dB)		SLL (dB)	Fabrication complexity		
	XZ-plane	YZ-plane	XZ-plane	YZ-plane				
^18^	44.3°	12.5°	< − 30	< − 60	< − 10	Complex		
^19^	Wide	14.6° (XY)	NA	NA	< − 10.9	Planar—Simple		
^20^	Wide	18.2°	NA	NA	< − 8	Planar—Simple		
^22^	18°**	18°**	NA	NA	< − 8	Complex		
^23^	25°**	30°**	NA	NA	< − 21	Multi-layer complex		
^24^	13°**	13°**	< − 30	< − 25	< − 7/− 8	Multi-layer Csomplex		
^25^	14°**	NA	NA	NA	< − 8	Complex		
^26^	12.9°	66.7°	< − 30	< − 70	< − 10	Complex		
Prop	09°—10°	Wide	< − 17.5	< − 30	< − 11	Planar—Simple		

Note: * indicate the approximate values. ** indicates the radiation efficiency.

The proposed array antenna, with wideband and high-gain characteristics, when integrated with software-defined radio, can be used for multi-band macro base station operation, enabling switching between frequency bands. It can also be used for point-to-point communication over short distances, indoor applications, integrated communication sensing, and many other uses. However, with the designed antenna, the phase of each element cannot be changed due to the fixed feed network; consequently, beam forming cannot be achieved with the current setup. However, with multiple such array structures and a beamforming network, the beam can be steered to certain degrees.

## Conclusion

This article presented a compact wideband millimeter-wave antenna designed using the CMT process for 5G wireless communication applications. The proposed single element was designed on an ultra-thin 0.254 mm substrate with a compact size of 8 × 6 mm^2^. The antenna is evolved in four stages; in each stage, the mode characteristics are studied in detail through modal significance, characteristic angles, modal-weighted coefficients, and surface current distribution. Each of these results depicted the mode behavior and the current orientation for comprehending the electric- or magnetic-dipole nature of each mode. Among these, the electric dipole modes are of paramount importance to the antennas linear polarization. Thus, by modifying the antenna geometry, the magnetic-dipole modes are suppressed, and the electric-dipole modes are excited across multiple frequency bands, thereby achieving a wide bandwidth. Furthermore, the antenna is expanded to an eight-element linear array to enhance the gain and directivity. The crucial challenge is to design the wideband feed network. This is achieved with a step-by-step approach: first designing a two-element array, then a four-element array, and finally an eight-element array with three stages of T-junction feed network. In the design of a feed network, the optimized length of the quarter-wave transformer plays a vital role in achieving a wideband response. Also, with the approximate half-wavelength spacing, the array antenna achieved a narrow beam, no grating lobes, and low SLL. Therefore, the proposed array antenna with its compact size, wide bandwidth, and high gain is a suitable candidate for future high-gain 5G mmWave wireless communication systems.

## Data Availability

All the data generated has been included in study.
